# Ceftriaxone- and N-acetylcysteine-induced brain tolerance to ischemia: Influence on glutamate levels in focal cerebral ischemia

**DOI:** 10.1371/journal.pone.0186243

**Published:** 2017-10-18

**Authors:** Weronika Krzyżanowska, Bartosz Pomierny, Beata Bystrowska, Lucyna Pomierny-Chamioło, Małgorzata Filip, Bogusława Budziszewska, Joanna Pera

**Affiliations:** 1 Department of Biochemical Toxicology, Faculty of Pharmacy, Jagiellonian University Medical College, Kraków, Poland; 2 Department of Toxicology, Faculty of Pharmacy, Jagiellonian University Medical College, Krakow, Poland; 3 Institute of Pharmacology, Polish Academy of Sciences, Laboratory of Drug Addiction Pharmacology, Kraków, Poland; 4 Department of Neurology, Faculty of Medicine, Jagiellonian University Medical College, Krakow, Poland; Indian Institute of Integrative Medicine CSIR, INDIA

## Abstract

One of the major players in the pathophysiology of cerebral ischemia is disrupted homeostasis of glutamatergic neurotransmission, resulting in elevated extracellular glutamate (Glu) concentrations and excitotoxicity-related cell death. In the brain, Glu concentrations are regulated by Glu transporters, including Glu transporter-1 (GLT-1) and cystine/Glu antiporter (system x_c_^-^). Modulation of these transporters by administration of ceftriaxone (CEF, 200 mg/kg, *i*.*p*.) or N-acetylcysteine (NAC, 150 mg/kg, *i*.*p*.) for 5 days before focal cerebral ischemia may induce brain tolerance to ischemia by significantly limiting stroke-related damage and normalizing Glu concentrations. In the present study, focal cerebral ischemia was induced by 90-minute middle cerebral artery occlusion (MCAO). We compared the effects of CEF and NAC pretreatment on Glu concentrations in extracellular fluid and cellular-specific expression of GLT-1 and xCT with the effects of two reference preconditioning methods, namely, ischemic preconditioning and chemical preconditioning in rats. Both CEF and NAC significantly reduced Glu levels in the frontal cortex and hippocampus during focal cerebral ischemia, and this decrease was comparable with the Glu level achieved with the reference preconditioning strategies. The results of immunofluorescence staining of GLT-1 and xCT on astrocytes, neurons and microglia accounted for the observed changes in extracellular Glu levels to a certain extent. Briefly, after MCAO, the expression of GLT-1 on astrocytes decreased, but pretreatment with CEF seemed to prevent this downregulation. In addition, every intervention used in this study seemed to reduce xCT expression on astrocytes and neurons. The results of this study indicate that modulation of Glu transporter expression may restore Glu homeostasis. Moreover, our results suggest that CEF and NAC may induce brain tolerance to ischemia by influencing GLT-1 and system x_c_^-^ expression levels. These transporters are presumably good targets for the development of novel therapies for brain ischemia.

## Introduction

Excitotoxicity related to excessive glutamate (Glu) release plays an important role in the pathophysiology of brain ischemia [1]. Glutamate transporter 1 (GLT-1) and cystine/glutamate antiporter (system x_c_^-^) are the principal proteins that cooperate in the active regulation of Glu extracellular concentrations [2,3]. GLT-1 transports Glu from the extracellular space to astrocytes, whereas system x_c_^-^ is implicated in the active transport of cystine to the intracellular space and reverse transport of Glu. Since brain ischemia is accompanied by a massive release of Glu, the recognition of the role of GLT-1 and system x_c_^-^ may to be crucial for understanding stroke pathophysiology and potential therapies. The glutamatergic system plays also a role in the development of brain tolerance to ischemia in response to short-lasting ischemia–a phenomenon called ischemic preconditioning (IP). Damaging brain ischemia decreases GLT-1 protein expression [4], whereas IP upregulates GLT-1 expression [5] and reduces ischemia-induced Glu release [6]. However, the effects of ischemia on system x_c_^-^ expression are still poorly characterized. Previously, we found that the expression of xCT mRNA decreased with no effect on protein levels 24 hours after focal cerebral ischemia [7], but there are also other inconsistent reports showing increased neuronal injury, following hypoxic-ischemic insults, associated with enhanced Glu excitotoxicity via increased function of the x_c_^-^ system. This neuronal injury was facilitated by IL-1b. [8]

Pharmacological modulation of GLT-1 by β-lactam antibiotics, including ceftriaxone (CEF), induces upregulation of GLT-1 expression and shows neuroprotective effects [9]. Intraperitoneal (*i*.*p*.) administration of CEF for five consecutive days prior to 90-minute middle cerebral artery occlusion (MCAO) has been shown to significantly reduce infarct volume and neurological deficits in rats [7,10].

N-acetylcysteine (NAC), a cysteine prodrug necessary for glutathione synthesis, can stimulate system x_c_^-^ activity [11]. In transient focal cerebral ischemia, NAC administered at the time of reperfusion significantly reduces infarct volume and levels of proinflammatory cytokines [12]. Repeated administration of NAC before transient ischemia significantly reduces infarct volume and expression of xCT protein [7]. System xc- is composed of two distinct subunits, xCT which is the light chain, catalytic protein, and 4F2hc which is the heavy chain protein, anchoring the light chain to the cellular membrane [3]. NAC has potent antioxidant properties, but it may also increase Glu concentration in the synaptic cleft and thus contribute to the progression of excitotoxicity.

Previously, we showed that CEF and NAC exert neuroprotective properties in the MCAO model (see Supporting Information figures: S13 Fig) [7]. CEF blunted downregulation of GLT-1 protein and mRNA, but also xCT mRNA however, not protein after cerebral ischemia (see Supporting Information figures: S14 and S15 Figs) [7]. NAC reduced the expression of xCT protein but not mRNA. These results are similar to the effects of two other well-recognized models of preconditioning, IP and chemical preconditioning with 3-nitropropionic acid (3NP). However, it is unclear, whether above changes evoked by CEF affect Glu level in brain ischemia. *In vitro* study reveals, that cells obtained from hippocampal CA1 field of animals pretreated with CEF either prior to SHAM-operation or global brain ischemia showed significantly increased Glu up-take. These effects were directly associated with GLT-1 activity [13]. Secondly, there is also no direct evidence that NAC reduces Glu levels during the time course of brain ischemia-reperfusion. This drug has been thought to reverse extracellular Glu elevations in psychotomimetic-induced models of schizophrenia [14]. Thus, mentioned results strongly suggest that the observed modulation of the studied Glu transporters, namely GLT-1 and xc- may be associated with changes in Glu levels during the time-course of brain ischemia-reperfusion.

In the current study, we continued to investigate the role of modulation of GLT-1 and xCT expression levels in the context of extracellular Glu levels in the ischemic brain and the cellular localization of Glu transporters. The present study is the first report about the fluctuation of Glu level in the extracellular fluid of the rat brain subjected to brain ischemic preconditioning, but also after treatment with CEF, NAC and 3NP. Since the mechanism of co-regulation of GLT-1 and xCT transporters is still poorly understood, the knowledge about the changes of Glu level in brain injury, neurodegenerative diseases, brain preconditioning is demanded.

## Methods

### Animals

All experiments were performed on male Wistar rats (280–320 g, Charles Rivers). The animals were maintained on a normal day-night cycle at 22±2°C with free access to food and water. All experimental protocols were in accordance with the Guide for the Care and Use of Laboratory Animals published by the National Institutes of Health and were approved by the First Local Ethical Committee at Jagiellonian University in Krakow (Permit No: 78/2011). All studies involving animals are reported according to the ARRIVE (Animal Research: Reporting of In Vivo Experiments) guidelines, including the blinding procedure of animal identities at each level of the experiment.

### Drugs and experimental design

CEF, NAC and 3NP were prepared in saline (the pH of the NAC solution was neutralized with 10% NaOH solution). The animals received one of the following solutions: CEF, NAC (200 mg/kg or 150 mg/kg body weight, respectively) or saline *i*.*p*. for five consecutive days. The last injection was administered 3 days prior to the 90-minute MCAO surgery or sham procedure. 3NP was administered in a single dose (20 mg/kg body weight) 3 days prior to 90-minute MCAO or sham surgery. The injection volume was 1 μL/g body weight.

The rats were randomly divided into the following ten groups of sixteen animals each: sham-operated rats receiving saline (SHAM), 90-minute MCAO rats receiving saline (ISCH), 20-minute MCAO 3 days prior to sham surgical procedure (IP), 20-minute MCAO 3 days prior to 90-minute MCAO (IP+MCAO), 3NP administration 3 days prior to sham surgical procedure (3NP), 3NP administration 3 days prior to 90-minute MCAO (3NP+MCAO), sham-operated rats receiving NAC (NAC), 90-minute MCAO rats receiving NAC (NAC+MCAO), sham-operated rats receiving CEF (CEF), and 90-minute MCAO receiving CEF (CEF+MCAO). Eight animals from each group were assigned to a microdialysis study, and the remaining eight rats were sacrificed for immunofluorescence staining.

### Implantation of microdialysis probe

Guide cannulas were implanted 24 hours prior to any surgical procedure. The rats were anesthetized with a ketamine/xylazine mixture (75 mg/kg ketamine, 5 mg/kg xylazine of body weight) administered *i*.*p*. The animals were stereotaxically implanted with guide cannulas (MAB 4; AgnTho’s, Stockholm, Sweden) aimed at the frontal cortex [anteroposterior (AP): -0.48 mm; mediolateral (ML): +2.0 mm; dorsoventral (DV): -1.2 mm] and the hippocampus (AP: -4.36 mm; ML: +1.8 mm; DV: -2.5 mm) according to the atlas of Paxinos & Watson (1998). The guide cannulas were affixed to the skulls with dental acrylic cement. Obturators were placed in the cannulas until the microdialysis probes were inserted.

### Microdialysis procedure

Microdialysis probes were perfused with artificial cerebrospinal fluid (aCSF) (in mM: NaCl 147, KCl 4.0, MgCl_2_ 1.0, CaCl_2_ 2.2, pH 7.4) at a constant flow rate (2 μL/minute) for two hours prior to collecting the first baseline sample. Next, the obturators were removed from the guide cannulas, and the microdialysis probes (MAB 4, membrane with a molecular weight 6-kDa cut-off, 2-mm length, 0.24-mm outer diameter, AgnTho’s AB, Lidingö, Sweden) were inserted into the guide cannulas. Next, the brain structures of animals dedicated to MCAO surgical procedures were perfused for 2 hours. After this period, samples were collected every 30 minutes as follows: two baseline samples, three samples during MCAO and two samples during the reperfusion period. All samples were immediately frozen and stored at -80°C until the LC-MS assay.

### Focal cerebral ischemia model

Middle cerebral artery occlusion was performed according to the method of Longa et al. [15] to induce transient focal cerebral ischemia. All surgical procedures were carried out under a stereoscopic microscope (MST 132 Lab 8, PZO Warszawa), and body temperature was maintained at a physiological level using a heating blanket (Homeothermic blanket system; Harvard Apparatus). The effectiveness of MCAO was confirmed using a blood flowmeter (PeriFlux System 5000; Perimed). Rats were anesthetized with a ketamine/xylazine solution (3/1, v/v, respectively) administered *i*.*p*. The doses of ketamine and xylazine were 75 mg/kg and 5 mg/kg body weight, respectively. The surgical procedure involved exposure of the left external carotid artery (ECA), the internal carotid artery (ICA) and the common carotid artery (CCA). All branches of the ECA were coagulated, and the artery was ligated and cut. The ICA and CCA were temporarily secured with microvascular clips. A silicone-coated filament (Doccol) was introduced into the lumen of the ECA and advanced until the blood flow dropped. The remaining clip on the CCA was removed, and the wound was secured with silk sutures. The occlusion times were 20 and 90 minutes for IP and MCAO, respectively. After the occlusion period, the wound was reopened, and the filament was removed to restore the blood flow. The wound was closed with sutures. The sham operation was carried out as described above, but the filament was not inserted.

### Liquid chromatography

The chromatographic separation was performed on an Agilent HPLC 1100 series system (Agilent, Waldbronn, Germany), which was equipped with a degasser, binary pump, autosampler and thermostated column compartment. The aCSF samples were separated on a LiChrospher 60 RP-select B column (125 mm x 4.6 mm ID, 5-μm particle size) in combination with an appropriate guard column (4 mm x 4 mm; 5-μm particle size) (Merck, Germany). All chromatographic separation parameters were used according to Jastrzębska et al. [16]. Samples were prepared by mixing 30 μL of the extracellular fluid with 4 μL of the internal standard Glu-d_5_ (25 μg/mL). The standard curve was plotted relating the glutamate concentration of standards to the peak area ratio of the glutamate to internal standard Glu-d_5_. The glutamate standard concentrations used for the preparation of the standard curve were as follows: 0, 25, 50, 100, 250, 500 and 1000 ng/mL of the extracellular fluid. Pairs of ions were monitored in the assay with the following values of m/z: 148.0/84.1 for Glu, 153.22/89.1 for Glu-d_5_. Data were analyzed using Analyst software 1.4 (AB SCIEX, USA). Levels of Glu were calculated using the calibration standard curves, which were constructed by linear regression analysis of peak area versus concentration curves.

### Tissue fixation

All rats were subjected to intracardiac 4% paraformaldehyde (PFA) perfusion 24 hours after the last surgical procedure. First, the animals were deeply anesthetized with ketamine (80 mg/kg) and xylazine (20 mg/kg). Then, the rats were transcardially perfused with 250 mL NaCl solution (0.9%; 32°C) until all remaining blood was removed. Next, the animals were perfused with 500 mL 4% PFA in 0.1 M phosphate-buffered saline (PBS). After the procedure, the brains were removed and maintained in the same 4% PFA solution overnight. Then, the brains were transferred to 0.1% sodium azide solution in 0.1 M PBS and stored until sectioning. Tissues were cut into coronal 50-μm sections using an automatic Vibratome (Leica VT1200 S) and stored in antifreezing medium solution (30% glycerol, 30% ethylene glycol in PBS).

### Immunohistochemistry—Double staining

All staining procedures, except for antigen retrieval, were conducted in sterile 24-well plates on a shaker. The sections were washed twice in PBS and transferred to glass vessels filled with tri sodium citrate buffer solution (pH≈9), which was used as an antigen retrieval buffer. The glass containers were closed with caps and moved to a water bath for 30 minutes at 80°C. Next, the containers were removed from the water bath and allowed to return to room temperature. The sections were then transferred to 24-well plates and washed twice in 0.2% Tween in PBS solution (PBST). Nonspecific antibody binding was blocked using 10% donkey normal serum (DNS) or 10% donkey and goat serum (1:1) (DNS+GNS) solution in PBST, depending on the combination of secondary antibodies. After a 1-hour incubation at room temperature, the blocking buffer was removed, and antibodies were added to the appropriate wells (antibodies and working dilutions are listed in Table 1). Results of specificity analysis of anti-xCT primary antibody are available in the Supplementary Information (S16 Fig). The slices were double-stained for GLT-1 or xCT together with neuronal marker (MAP2) or astroglial marker (GFAP) or microglial marker (Iba1). Each mixture of two specific antibodies was dissolved in an appropriate 2% DNS or DNS+GNS serum in PBST. Then, the 24-well plates were placed in fridge at 4°C overnight. The following day, the plates were removed from the fridge and incubated for one hour on shaker at room temperature. Next, the sections were washed twice in 2% DNS or DNS+GNS in PBST, and secondary antibodies dissolved in PBST were added to the appropriate wells and incubated for 1 hour in the dark at room temperature. Finally, the sections were washed three times in PBST, transferred to microscopic glass slides, dried, mounted in Fluoroshield mounting medium (Sigma) and coverslipped. The staining was visualized using a Leica DMIL fluorescence inverted microscope. Images of motor-frontal cortex and CA1 field of the hippocampus were captured by a digital CCD camera (Leica DFC3000G).

**Table 1:** The list of specific primary and secondary antibodies as well as sera used in immunofluorescence staining with the appropriate dilutions.

Primary antibodies	Secondary antibodies	Serum
Anti-xCT rabbit (ab37185 Abcam) dilution: 1:200	Donkey anti-rabbit TR (ab6800 Abcam) dilution: 1:1000	Donkey normal serum (ab7475 Abcam) dilution: 10%
Anti-EAAT2 mouse (sc135892 Santa Cruz Biotechnology) dilution: 1:100	Donkey anti-mouse TR (sc2785 Santa Cruz Biotechnology) dilution: 1:300	Goat normal serum (50062Z Thermo Fisher Scientific) dilution: 10%
Anti-GFAP goat (sc6170 Santa Cruz Biotechnology) dilution: 1:100	Donkey anti-goat FITC (sc2024 Santa Cruz Biotechnology) dilution: 1:300	
Anti-Iba1 goat (ab5076 Abcam) dilution: 1:200	Goat anti-chicken Alexa Fluor 488 (A11039 Thermo Fisher Scientific) dilution: 1:300	
Anti-MAP2 chicken (ab5392 Abcam) dilution: 1:1000		

### Statistical analysis

Microdialysis results were analyzed by 2-way repeated measures ANOVA with Sidak post hoc test. Each statistical analysis compared four experimental groups: intervention alone, intervention + MCAO, SHAM and MCAO. Data are presented as the mean ± S.E.M. A probability value of p < 0.05 was considered significant.

## Results

### Extracellular levels of glutamate

**Fig 1:**
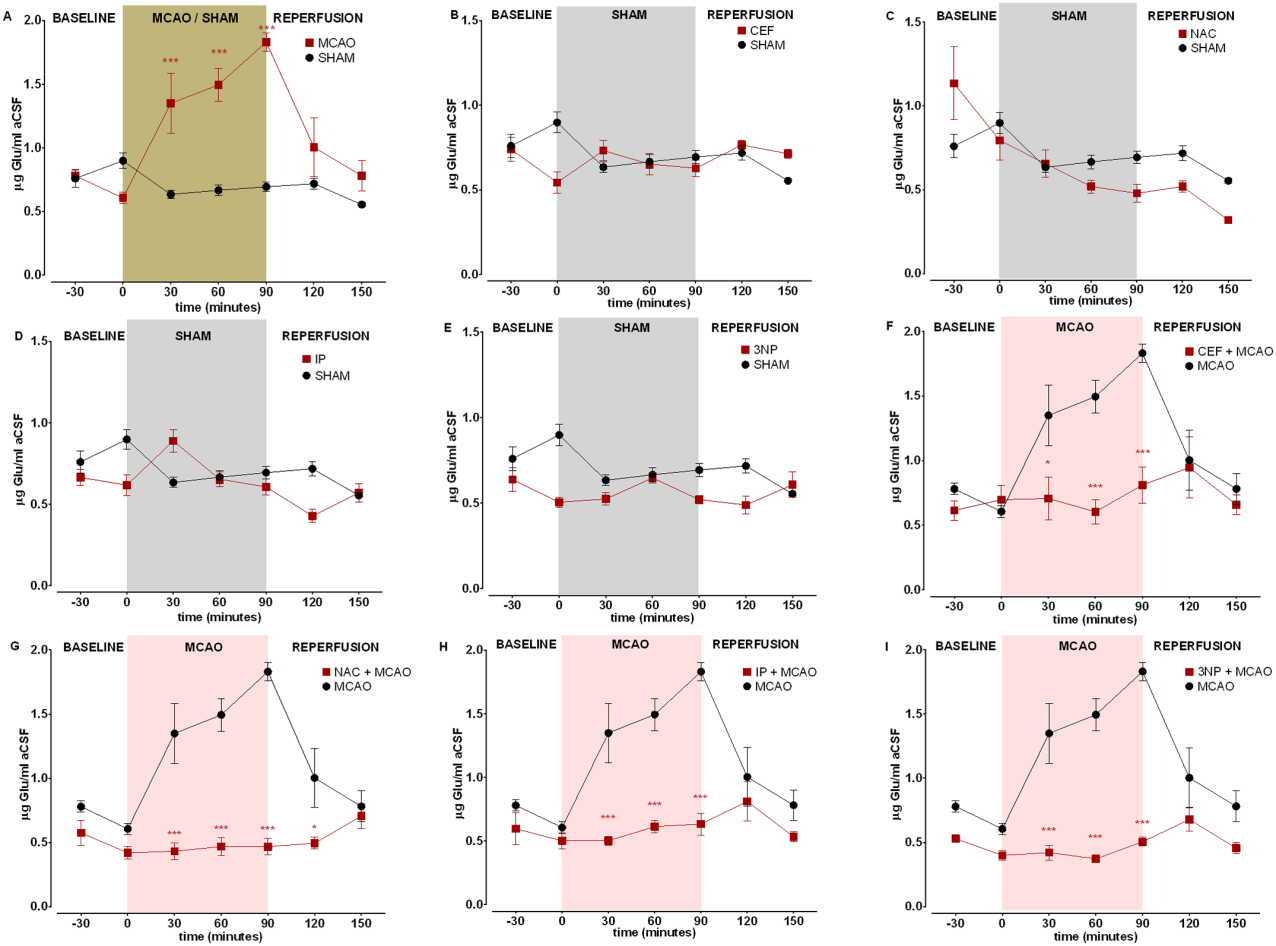
Glu concentration changes in dialysate samples during brain ischemia/reperfusion in the frontal cortex. The level of Glu is expressed as μg/mL aCSF. Data are presented as the mean ± SEM (n = 8). Two-way repeated measures ANOVA was conducted, followed by Sidak *post hoc* test (*p<0.05, **p <0.01, ***p<0.001 compared with ISCH or SHAM). Microdialysates were collected during the following periods: preischemia (-30 to 0 minutes); ischemia (0–90 minutes) and reperfusion (90–150 minutes). (A) MCAO vs SHAM, (B) CEF vs SHAM, (C) NAC vs SHAM (D) IP vs SHAM, (E) 3NP vs SHAM, (F) CEF + MCAO vs MCAO, (G) NAC + MCAO vs MCAO, (H) IP + MCAO vs MCAO, (I) 3NP + MCAO vs MCAO.

**Fig 2:**
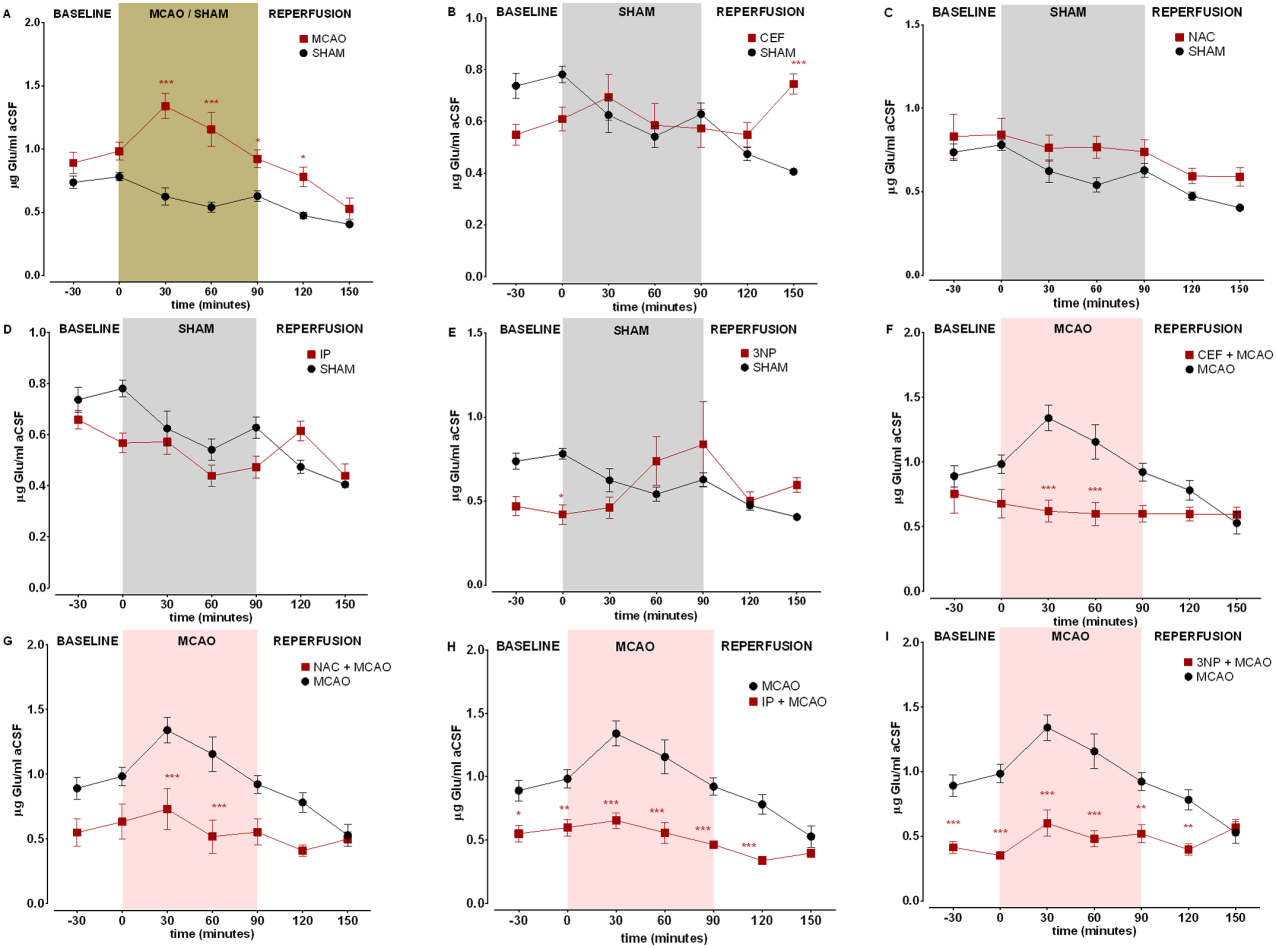
Glu concentration changes in dialysate samples during brain ischemia/reperfusion in the hippocampus. The levels of Glu are expressed as μg/mL aCSF. Data are presented as the mean ± SEM (n = 8). Two-way repeated measures ANOVA was conducted, followed by Sidak *post hoc* test (*p<0.05, **p <0.01, ***p<0.001 compared with ISCH or SHAM). Microdialysates were collected during the following periods: preischemia (-30 to 0 minutes); ischemia (0–90 minutes) and reperfusion (90–150 minutes). (A) MCAO vs SHAM, (B) CEF vs SHAM, (C) NAC vs SHAM (D) IP vs SHAM, (E) 3NP vs SHAM, (F) CEF + MCAO vs MCAO, (G) NAC + MCAO vs MCAO, (H) IP + MCAO vs MCAO, (I) 3NP + MCAO vs MCAO.

In the MCAO group, a dramatic increase in Glu levels was observed in both the frontal cortex and hippocampus at 30, 60 and 90 minutes of occlusion. During the first 60 minutes of reperfusion, the Glu levels decreased and were similar to those in the MCAO and SHAM groups in the frontal cortex. In the hippocampus at 30 minutes of reperfusion, significantly increased Glu concentrations were recorded in the MCAO group (Figs 1A and 2A).

Two-way ANOVA revealed that the level of Glu during ischemia was significantly lower (at 30 minutes after occlusion p < 0.05 at 60 minutes and 90 minutes after occlusion, p < 0.001) in the frontal cortex of animals pretreated with CEF than that in non-pretreated animals (Fig 1F). A similar reduction was observed in the hippocampus (at 30 minutes and 60 minutes after occlusion p < 0.001) but not at the end of ischemia, when no significant differences were recorded (Fig 2F). Glu concentrations at baseline and during reperfusion were similar in both groups (CEF+MCAO and MCAO) and both brain structures.

As shown by two-way ANOVA, NAC pretreatment significantly decreased Glu concentration during the entire ischemia period in the frontal cortex (p < 0.001) (Fig 1G), whereas this effect was observed at only 30 and 60 minutes after occlusion in the hippocampus (p < 0.001) and not at 90 minutes (Fig 2G). Moreover, in the frontal cortex of NAC-pretreated animals, the Glu concentration remained significantly lower (p < 0.05) within the first 30 minutes of reperfusion.

In the IP group, two-way ANOVA showed significantly reduced Glu concentrations in the frontal cortex and hippocampus at all three occlusion time points (p < 0.001) (Fig 1H). Interestingly, the baseline Glu level was significantly lower (p < 0.05) in the hippocampus, and this reduction was also observed at the beginning of reperfusion (p < 0.001) (Fig 2H).

Preconditioning with 3NP significantly reduced Glu concentrations in the frontal cortex during ischemia (p < 0.001), with no influence on the baseline and reperfusion periods (Fig 1I). Whereas in the hippocampus of animals pretreated with 3NP, Glu reduction was observed not only during occlusion (at 30 and 60 minutes p < 0.001, at 90 minutes p < 0.01) but also at baseline (p < 0.001) compared with that in the MCAO group. This reduction was also present within the first 30 minutes of reperfusion (p < 0.01) (Fig 2I).

### Immunofluorescence staining of brain slices

Merged images of studied slices showed expression of GLT-1 on astrocytes (GFAP), but there was no expression of this transporter on neurons (MAP-2) or microglia (Iba1). Expression of xCT was visible on all studied cells.

**Fig 3:**
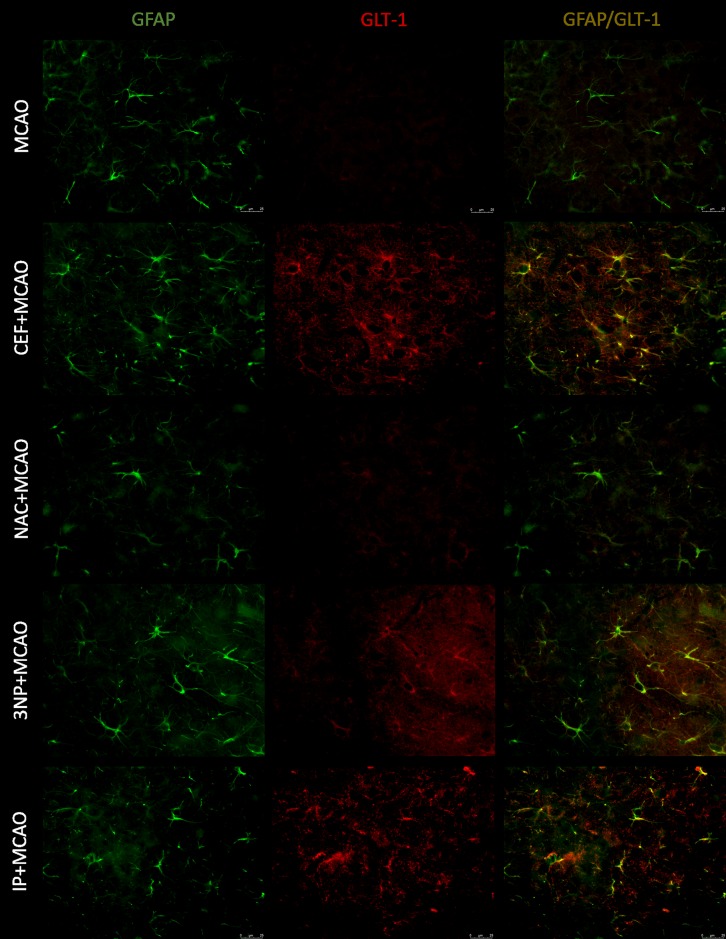
Expression of GLT-1 on astrocytes within motor-frontal cortex. GLT-1 (Texas Red, red) and GFAP (FITC, green) double staining of brain sections of animals subjected to 90-minute MCAO, preceded by preconditioning. Scale bars represent 25 μm, for each group n = 8.

**Fig 4:**
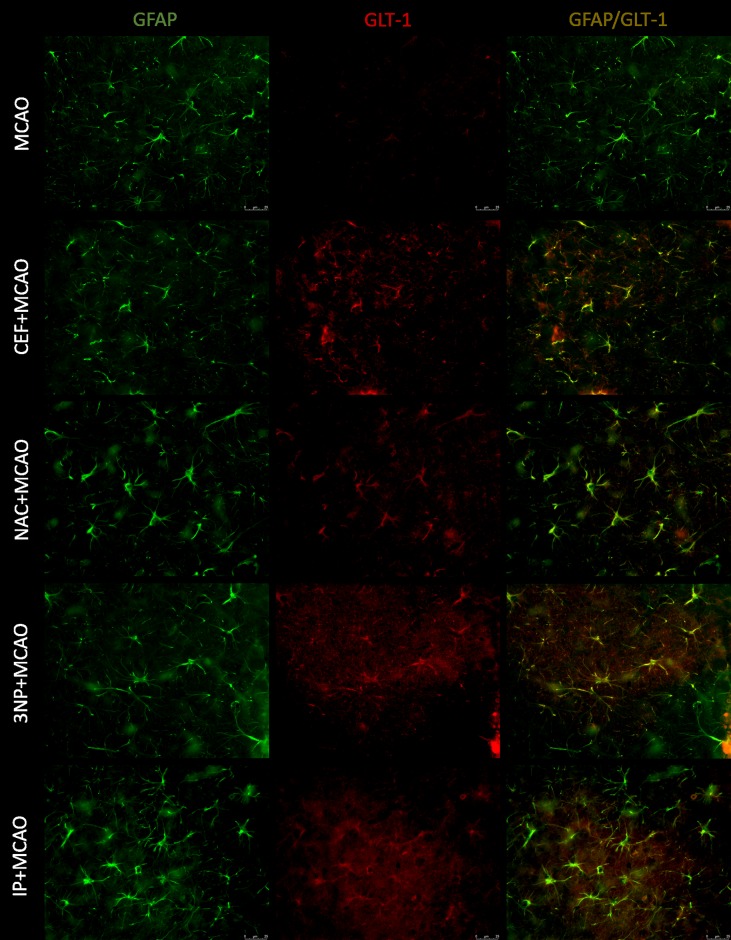
Expression of GLT-1 on astrocytes within CA1 field of hippocampus. GLT-1 (Texas Red, red) and GFAP (FITC, green) double staining of brain sections of animals subjected to 90-minute MCAO, preceded by preconditioning. Scale bars represent 25 μm, for each group n = 8.

Pretreatment with CEF seemed to prevent downregulation of GLT-1 on astrocytes after 90-minute ischemia in both the frontal cortex and hippocampus (Figs 3 & 4). Similar results were seen in the brains of rats subjected to 90-minute MCAO preceded by IP or 3NP. Similar to our previously published ELISA results [7] (see S14 Fig), we did not observe notable effects of NAC treatment on GLT-1 expression in any brain structure. For SHAM groups, see figure in S1 Fig and figure in S2 Fig.

GFAP and xCT double staining showed that every intervention used resulted in diminished xCT expression in astrocytes in the frontal cortex and hippocampus after prolonged ischemia (figures in S3–S6 Figs).

**Fig 5:**
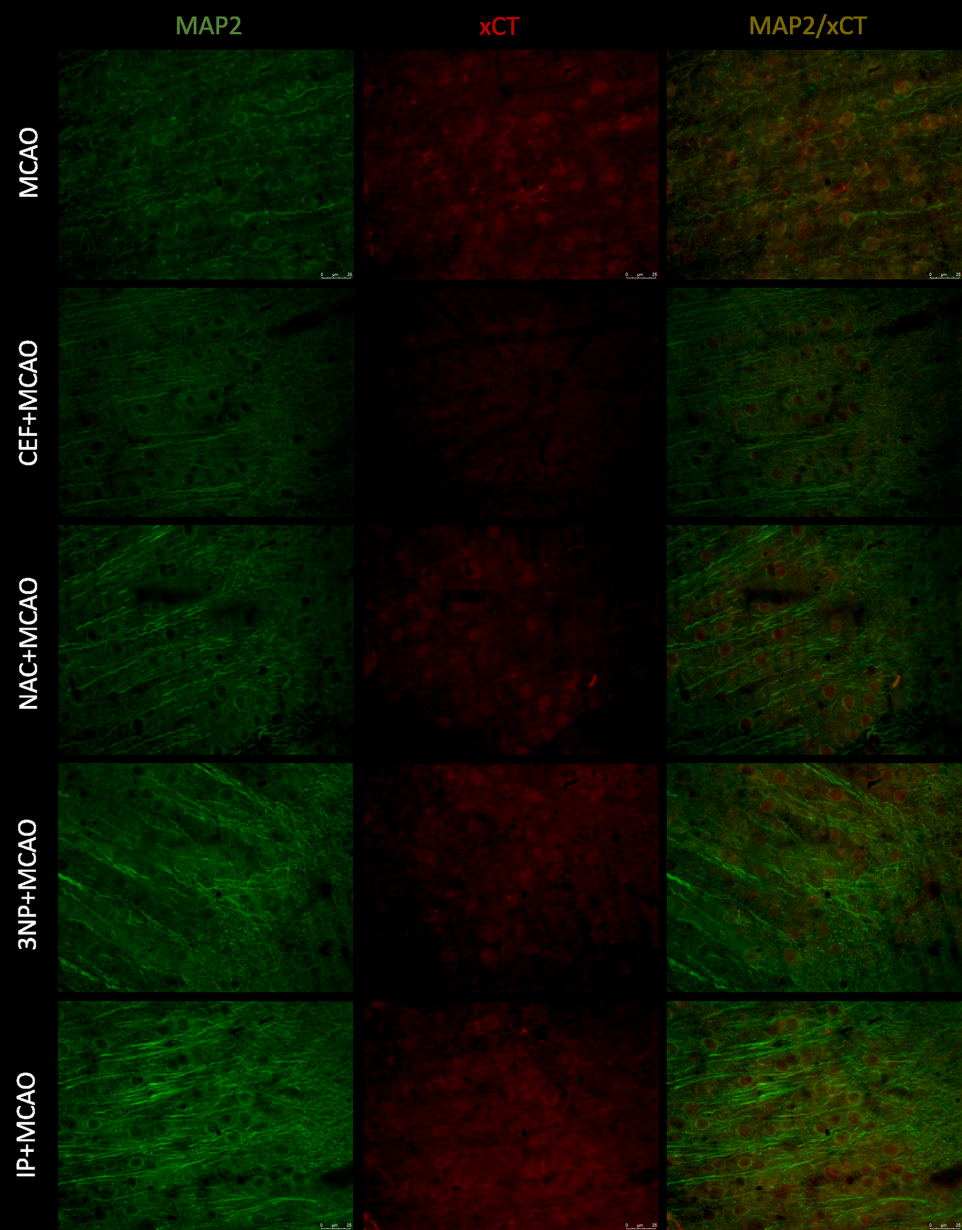
Expression of xCT on neuronal cells within the motor-frontal cortex. xCT (Texas Red, red) and MAP2 (Alexa Fluor 488, green) double staining of brain sections of animals subjected to 90-minute MCAO, preceded by preconditioning. Scale bars represent 25 μm, for each group n = 8.

**Fig 6:**
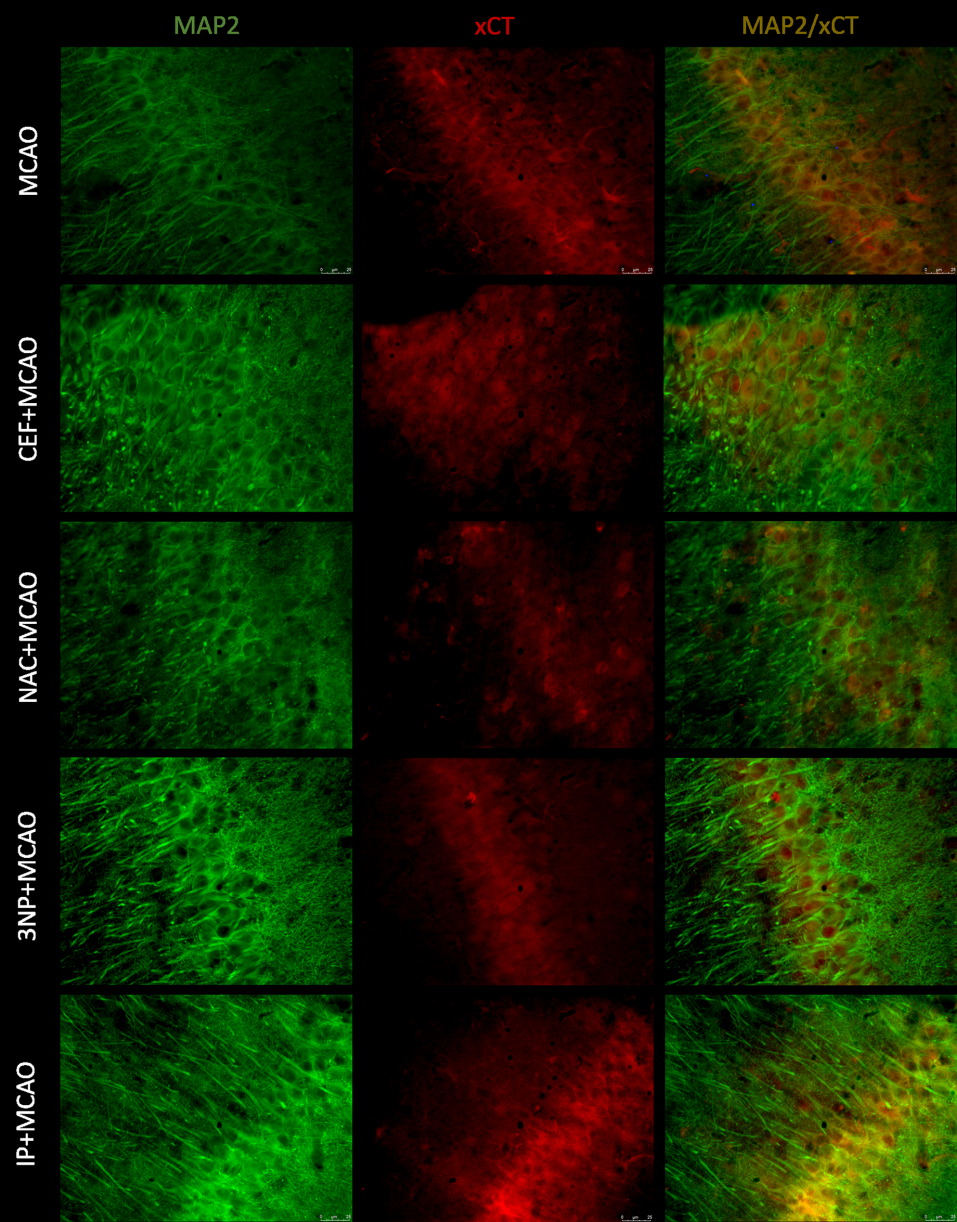
Expression of xCT on neuronal cells within the CA1 field of the hippocampus. xCT (Texas Red, red) and MAP2 (Alexa Fluor 488, green) double staining of brain sections of animals subjected to 90-minute MCAO, preceded by preconditioning. Scale bars represent 25 μm, for each group n = 8.

Only in animals pretreated with CEF, the results of MAP2 staining suggested that neuronal expression of xCT was reduced in the frontal cortex but not in the hippocampus. In the remaining groups of animals subjected to ischemia, the coexpression of MAP2/xCT was comparable to that of the MCAO group (Figs 5 & 6). SHAM groups are presented in figure in S7 Fig and figure in S8 Fig.

The expression of xCT was found only on microglia of animals pretreated with 3NP 72 hours before ischemia. In the remaining groups subjected to ischemia, the coexpression of Iba1/xCT was inconspicuous (figures in S9–S12 Figs).

## Discussion

Excitotoxicity is the first and the most intensively studied molecular mechanism of cerebral ischemia. This process is based on the massive release of Glu, overstimulation of glutaminergic receptors with simultaneous failure of reuptake mechanisms. Excessive amount of Glu activates many intracellular pathways, what inter alia cause increased calcium influx, activation of protein kinases, generation of reactive oxygen species. Because all these processes are demanding energy, exicitotoxicity results in the ATP depletion, loss of membrane potential, dysfunction of the respiratory chain and activation of various cell death pathways, including apoptosis and necrosis. Excitotoxicity of glutamate is a key player in neurodegenerative diseases such as amyotrophic lateral sclerosis, Azheimer’s disease and Parkinson’s disease. The pathological role of Glu is also crucial in stroke and traumatic brain injury-induced neurodegeneration [17]. Numerous clinical trials have focused on the inhibition of glutamate-mediated neurotoxicity in many various central nervous system diseases including brain ischemia, however with no success. Brain preconditioning which induce brain tolerance to ischemia brings new hope for the development of new therapeutic strategies of clinical relevance. The mechanism of this phenomenon is not fully explained however, it has been shown it involves glutamatergic system modulation [18]. The aim of brain preconditioning is to reduce glutamate excitotoxicity, however still there is no pharmacological approach to induce this phenomenon. Our team is focused on the search for the pharmacological agents, e.g. CEF or NAC, inducing brain tolerance to ischemia (see S13 Fig).

The results of the current study confirmed that 90-minute MCAO caused a significant increase in the extracellular Glu levels in the frontal cortex and hippocampus [19,20]. This finding may be associated with reduced expression of GLT-1 after prolonged cerebral ischemia. The results of our previous study showed a significant downregulation of GLT-1 in the frontal cortex and hippocampus at both the mRNA and protein levels following cerebral ischemia [7] (see S14 Fig). Similar conclusions were drawn by Zhang et al. [21]. Immunohistochemically, weaker expression of GLT-1 on astrocytes has been shown in the hippocampal CA1 field of animals subjected to ischemic insult [21].

In the present study, we used two different approaches, namely, IP and 3NP, which are known methods that can induce brain tolerance to ischemia. IP involves multidirectional molecular changes, and modulation of the glutamatergic system is one of its most important elements [22]. For instance, mild activation of the NMDA receptor is involved in the development of brain tolerance to ischemia during IP [23]. Moreover, downregulation of GLT-1 protein expression induced by severe brain ischemia was prevented by IP [7,21].

Here, IP that was performed 3 days prior to 90-minute MCAO significantly lowered Glu levels compared with those in rats subjected to ischemia alone in both the frontal cortex and hippocampus during ischemia. Gong et al. [24] have also shown that IP significantly attenuates increased Glu concentrations during global brain ischemia in rats. These changes were directly associated with GLT-1 activity. Pre-administration of dihydrokainate, a GLT-1 inhibitor, diminishes the protective effects of IP and sustains the elevated Glu levels during brain ischemia. Gong et al. [24] have also found that induction of tolerance to brain ischemia through IP is associated with upregulation of GLT-1 expression. These findings are consistent with our present report, which showed that the development of tolerance to brain ischemia induced by IP is accompanied by attenuation of reduced GLT-1 expression that occurs after 90-minute MCAO [7]. The immunofluorescence staining results showed strong coexpression of GLT-1 and GFAP in both the frontal cortex and CA1 field of the hippocampus. This finding suggests that modulation of the GLT-1 levels in astrocytes is responsible for restoration of proper Glu clearance in animals that underwent IP prior to brain ischemia. In addition, the staining showed weaker coexpression of xCT and GFAP in animals that underwent IP, and this result correlates with reduced Glu levels during prolonged ischemia.

The second preconditioning strategy used in this study was administration of 3NP—an irreversible inhibitor of succinate dehydrogenase activity, an enzyme involved in the mitochondrial respiratory chain [25]. 3NP has been shown to activate NF-κB, a transcription factor [26] that promotes GLT-1 expression [27]. The Glu level following administration of 3NP has not been investigated in the MCAO animal model. In the present study, administration of 3NP prior to 90-minute brain ischemia significantly reduced Glu levels in samples collected during MCAO in the frontal cortex and hippocampus. Moreover, in the hippocampus, decreased Glu levels were also found during the preischemic period as well as during the initial phase of reperfusion. These results may also be associated with increased GLT-1 protein levels in animals pretreated with 3NP. Previously, we found that this preconditioning strategy attenuated decreased GLT-1 protein expression in severe brain ischemia in a similar manner to IP [7]. Moreover, brain sections from animals that received 3NP prior to severe ischemia showed stronger expression of GLT-1 on astrocytes in the frontal cortex and CA1 field of the hippocampus comparing with that in non-preconditioned animals. Similar to IP rats, 3NP pretreatment also reduced the expression of xCT on astrocytes in both examined brain structures.

In reference to these recognized preconditioning strategies, we studied the effects of CEF and NAC as potential inducers of brain tolerance to ischemia. Ceftriaxone, a beta-lactam antibiotic, is known to induce GLT-1 expression. The neuroprotective effects of CEF have been shown *in vitro* [9]. Moreover, CEF has demonstrated neuroprotective activity in several ischemia models, including focal cerebral ischemia induced by MCAO and global ischemia [7,10,28–31]. These effects of CEF have been linked to GLT-1 upregulation [10,30,31]. In the current study, we showed that these protective effects of CEF administration were associated with significantly lower Glu levels in aCSF samples collected during ischemia in both the frontal cortex and hippocampus. Moreover, we have previously demonstrated that CEF administration inhibits GLT-1 protein downregulation caused by severe ischemia [7]. These findings are consistent with our immunofluorescence staining. Specifically, we observed stronger GLT-1 expression on astrocytes in both the frontal cortex and CA1 field of the hippocampus. All these changes are comparable to the effects obtained by IP with 3NP used as the preconditioning strategy. The immunofluorescence results also indicate reduced expression of xCT on astrocytes in both the frontal cortex and CA1 field of the hippocampus of animals pretreated with CEF prior to ischemia. This finding may suggest that decreased xCT expression is associated with reduced extracellular Glu levels during MCAO.

It is worth to mention, that the most current data indicate that CEF may not only increase GLT-1, but also xCT expression. Knackstedt and co-workers (2010) showed, that CEF attenuation of reinstatement in rats, trained to self-administer cocaine, involves upregulation of both GLT-1 and xCT protein expression in nucleus accumbens [32]. It was suggested, that this xCT up-regulation, may lead to an increased non-synaptic glutamate efflux via xc- system. This in turn may stimulate extrasynaptic mGluR2/3 and in consequence reduce synaptic glutamate release [33]. Employing antisense strategy to knock-down GLT-1 expression in ceftriaxone inhibition of reinstatement in cocaine addiction, prevented from upregulation of xCT. Similarly, xCT knockdown prevented from GLT-1 expression up-regulation in the same model. This intriguing phenomenon shows a common regulatory mechanism for GLT-1 and xCT expression. It has been claimed, that at least in that experimental design the action of ceftriaxone, leading to up-regulation of both GLT-1 and xCT transporters expression does not involve transcriptional mechanism [34]. Also, up-regulation of xCT expression at the mRNA level as well as an increase in xc- activity was found in HT22 cells pre-treated with CEF for 7 days before exposure to toxic concentration of glutamate. These cells also showed higher expression of nuclear factor erythroid 2-related factor 2 (Nrf2) at the protein level, which activates transcription of xCT gene. Thus, this increase in Nrf2 protein level, is the probable mechanism of CEF-stimulated xCT expression up-regulation in this in vitro model [35].

The second pharmacological attempt to induce brain tolerance to ischemia was 5-day administration of NAC. Similar to CEF, treatment with NAC significantly lowered the level of Glu in aCSF obtained during brain ischemia in the frontal cortex and hippocampus. NAC is a glutathione synthesis substrate and thus promotes free radical species scavenging and cell survival. Thus, the simplest explanation for the reduced Glu levels in animals that were exposed to NAC before ischemia could be the reduction of oxidative stress-induced damage that promotes excitotoxicity [36]. Although NAC has been shown to upregulate expression of GLT-1 [37], it seems unlikely that the above changes are associated with modulation of this Glu transporter. Immunofluorescence GLT-1 staining in both the frontal cortex and hippocampus of animals pretreated with NAC did not differ from the results seen in animals that underwent 90-minute MCAO alone. Additionally, in our previous study, administration of NAC did not change GLT-1 mRNA and protein expression after brain ischemia [7]. On the other hand, we cannot exclude the possibility that the observed decrease in Glu levels after NAC administration in animals that underwent brain ischemia may be due to modulation of system x_c_^-^ expression. Previously, we have found a significant reduction in xCT protein expression following administration of NAC in animals that underwent 90-minute MCAO, as well as in control rats (see S15 Fig) [7]. The immunofluorescence staining in this study showed that xCT expression on astrocytes could be reduced in the analyzed brain regions. However, the xCT expression in neuronal cells seemed to be stronger in animals pretreated with NAC. This finding could suggest different regulation of xCT expression depending on the cell type. Under conditions with compromised Glu uptake (like ischemia), increased Il-1β has been shown to enhance the expression/activity of system x_c_^-^ only on astrocytes, which contributes to increased excitotoxicity [38]. Preconditioning strategies, such as IP and 3NP, are known to reduce Il-1β expression, and this may explain the obtained results for xCT and GFAP coexpression [39]. There are also data confirming similar influences of NAC on the expression of Il-1β [12]. Thus, one can speculate that the interventions used in this study caused changes in the expression of xCT but only within astrocytes. However, we did not perform quantitative analysis of the immunofluorescence staining of transporter expression.

In conclusion, the results of the current study showed the protective effects of two pharmacological strategies, namely, CEF and NAC, which were demonstrated by reduction of Glu levels during severe brain ischemia, resembling the effects of the two reference preconditioning methods, IP and 3NP. Changes in the expression levels of xCT and GLT-1 are cell-type specific and mirror the neuroprotective properties of the studied compounds.

## Supporting information

S1 Fig Expression of GLT-1 on astrocytes within motor-frontal cortex of sham animals. GLT-1 (Texas Red, red) and GFAP (FITC, green) double staining of brain sections of animals subjected to sham surgery, preceded by preconditioning. Scale bars represent 25 μm, for each group n = 8.
(TIF)

S2 Fig Expression of GLT-1 on astrocytes within CA1 field of hippocampus of sham animals. GLT-1 (Texas Red, red) and GFAP (FITC, green) double staining of brain sections of animals subjected to sham surgery, preceded by preconditioning. Scale bars represent 25 μm, for each group n = 8.
(TIF)

S3 Fig Expression of xCT on astrocytes within motor-frontal cortex of animals subjected to brain ischemia. xCT (Texas Red, red) and GFAP (FITC, green) double staining of brain sections of animals subjected to 90-minute MCAO, preceded by preconditioning. Scale bars represent 10 μm, for each group n = 8.
(TIF)

S4 Fig Expression of xCT on astrocytes within motor-frontal cortex of sham animals. xCT (Texas Red, red) and GFAP (FITC, green) double staining of brain sections of animals subjected to sham surgery, preceded by preconditioning. Scale bars represent 10 μm, for each group n = 8.
(TIF)

S5 Fig Expression of xCT on astrocytes within CA1 field of hippocampus of animals subjected to brain ischemia. xCT (Texas Red, red) and GFAP (FITC, green) double staining of brain sections of animals subjected to 90-minute MCAO, preceded by preconditioning. Scale bars represent 10 μm, for each group n = 8.
(TIF)

S6 Fig Expression of xCT on astrocytes within CA1 field of hippocampus of sham animals. xCT- (Texas Red, red) and GFAP (FITC, green) double staining of brain sections of animals subjected to sham surgery, preceded by preconditioning. Scale bars represent 10 μm, for each group n = 8.
(TIF)

S7 Fig Expression of xCT on neuronal cells within motor-frontal cortex of sham animals. xCT (Texas Red, red) and MAP2 (Alexa Fluor 488, green) double staining of brain sections of animals subjected to sham surgery, preceded by preconditioning. Scale bars represent 25 μm, for each group n = 8.
(TIF)

S8 Fig Expression of xCT on neuronal cells within CA1 field of hippocampus of sham animals. xCT (Texas Red, red) and MAP2 (Alexa Fluor 488, green) double staining of brain sections of animals subjected to sham surgery, preceded by preconditioning. Scale bars represent 25 μm, for each group n = 8.
(TIF)

S9 Fig Expression of xCT on microglia within motor-frontal cortex of animals subjected to brain ischemia. xCT (Texas Red, red) and Iba1 (FITC, green) double staining of brain sections of animals subjected to 90-minute MCAO, preceded by preconditioning. Scale bars represent 10 μm, for each group n = 8.
(TIF)

S10 Fig Expression of xCT on microglia within motor-frontal cortex of sham animals. xCT (Texas Red, red) and Iba1 (FITC, green) double staining of brain sections of animals subjected to sham surgery, preceded by preconditioning. Scale bars represent 10 μm, for each group n = 8.
(TIF)

S11 Fig Expression of CT on microglia within CA1 field of hippocampus of animals subjected to brain ischemia. xCT (Texas Red, red) and Iba1 (FITC, green) double staining of brain sections of animals subjected to 90-minute MCAO, preceded by preconditioning. Scale bars represent 10 μm, for each group n = 8.
(TIF)

S12 Fig Expression of xCT on microglia within CA1 field of hippocampus of sham animals. xCT (Texas Red, red) and Iba1 (FITC, green) double staining of brain sections of animals subjected to sham surgery, preceded by preconditioning. Scale bars represent 10 μm, for each group n = 8.
(TIF)

S13 Fig Neuroprotective effects of used interventions. **(A)** decreased neurological deficit 24 h after ischemia (***p<0.001 vs. ISCH, *Mann-Whitney*, n = 24/group). **(B)** representative TTC-stained brain sections and corresponding histogram **(C)** representing calculated infarct volume in VEH+ISCH, CEF+ISCH, NAC+ISCH, 3NP+ISCH, and IP+ISCH groups (***p<0.001 vs. VEH+ISCH, *t*-test, n = 8/group) 24 h after reperfusion. The figure obtained from our previous manuscript entitled: ‘*N*-Acetylcysteine and Ceftriaxone as Preconditioning Strategies in Focal Brain Ischemia: Influence on Glutamate Transporters Expression’, Neurotoxicity Research, 2016; 29:539–550 under the terms of the Creative Commons Attribution 4.0 International License (http://creativecommons.org/licenses/by/4.0/).
(TIF)

S14 Fig Influence of used interventions on expression of mRNA (A) and protein (B) of GLT-1 in frontal cortex (FC), hippocampus (HIP) and dorsal striatum (DS) in studied groups: VEH+SHAM, VEH+ISCH, CEF+SHAM, CEF+ISCH, NAC+SHAM, NAC+ISCH, 3NP+SHAM, 3NP+ISCH, IP+ISCH, and IP+SHAM (*p<0.05, **p<0.01, ***p<0.001 vs VEH+ISCH or VEH+SHAM, Student’s *t*-test, #p<0.05, ##p<0.01, ###p<0.001 vs VEH+ISCH or VEH+SHAM, Two-way ANOVA, Post-hoc Tukey’s test, n = 8/group) 24 h after reperfusion. The figure obtained from our previous manuscript entitled: ‘*N*-Acetylcysteine and Ceftriaxone as Preconditioning Strategies in Focal Brain Ischemia: Influence on Glutamate Transporters Expression’, Neurotoxicity Research, 2016; 29:539–550 under the terms of the Creative Commons Attribution 4.0 International License (http://creativecommons.org/licenses/by/4.0/).
(TIF)

S15 Fig Influence of used interventions on expression of mRNA (A) and protein (B) of xc- in frontal cortex (FC), hippocampus (HIP) and dorsal striatum (DS) in studied groups: VEH+SHAM, VEH+ISCH, CEF+SHAM, CEF+ISCH, NAC+SHAM, NAC+ISCH, 3NP+SHAM, 3NP+ISCH, IP+ISCH, and IP+SHAM (*p<0.05, **p<0.01, ***p<0.001 vs VEH+ISCH or VEH+SHAM, Student’s *t*-test, #p<0.05, ##p<0.01, ###p<0.001 vs VEH+ISCH or VEH+SHAM, Two-way ANOVA, Post-hoc Tukey’s test, n = 8/group) 24 h after reperfusion. The figure obtained from our previous manuscript entitled: ‘*N*-Acetylcysteine and Ceftriaxone as Preconditioning Strategies in Focal Brain Ischemia: Influence on Glutamate Transporters Expression’, Neurotoxicity Research, 2016; 29:539–550 under the terms of the Creative Commons Attribution 4.0 International License (http://creativecommons.org/licenses/by/4.0/).
(TIF)

S16 Fig Confirmation of the specificity of primary antibody against xCT and the role of antigen retrieval for the proper antibody binding. **1 & 2**: double immunofluorescent staining with or without antigen retrieval. **3 & 4**: negative staining control with the omission in the procedure adding of primary antibody against xCT, MAP2 or both antibodies. **5A**: An exemplary immunoblot membrane obtained using the same antibody anti-xCT as in immunofluorescent staining. **5B**: A positive control staining of brain meninges, where expression of system x_c_^-^ is known to be high.
(TIF)
