# Supplementary figures and images for: Ceftriaxone- and N-acetylcysteine-induced brain tolerance to ischemia: Influence on glutamate levels in focal cerebral ischemia
Source: PLoS One. 2017 Oct 18;12(10):e0186243. doi: 10.1371/journal.pone.0186243 (PMC5646803; doi:10.1371/journal.pone.0186243)

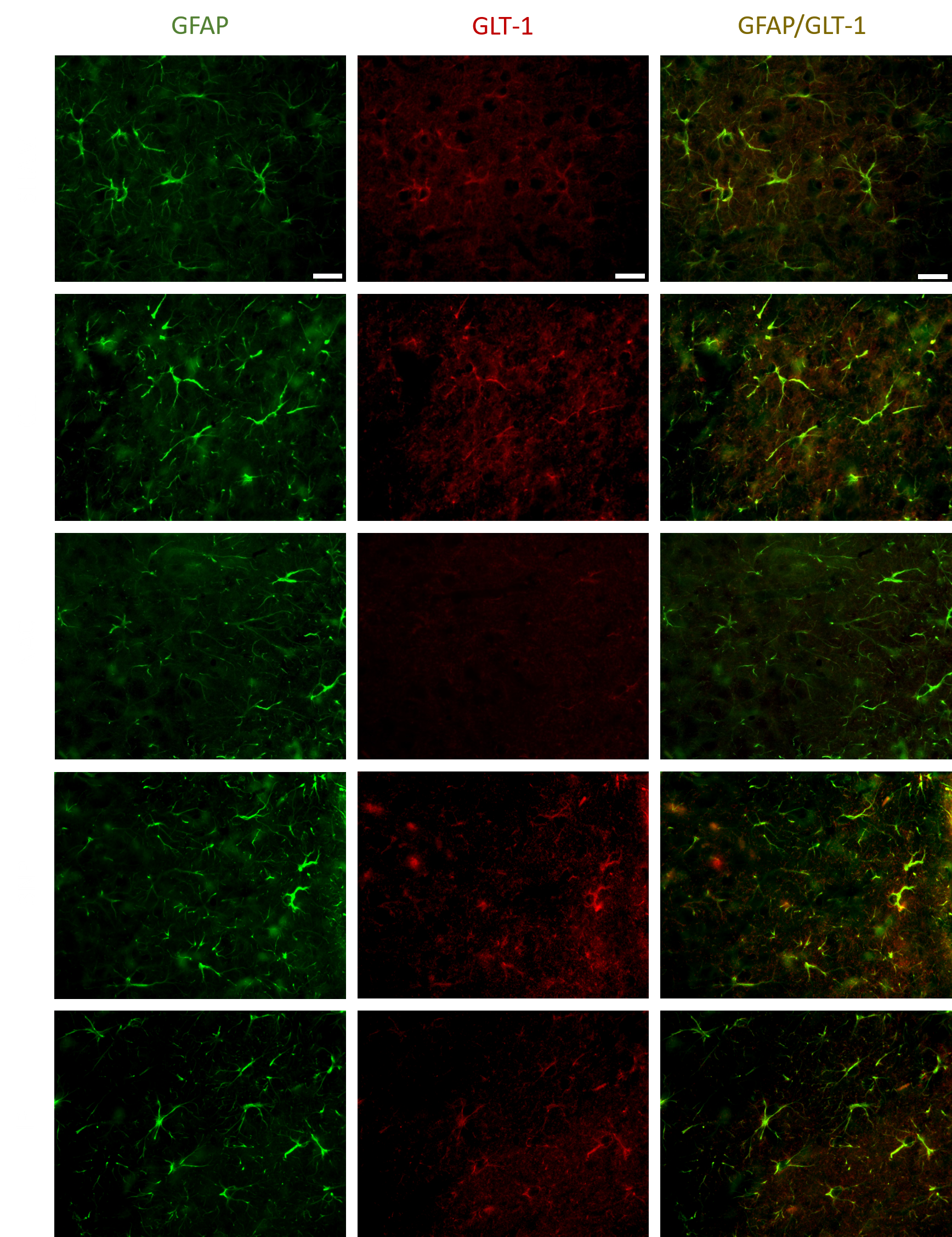

Supplement: S1 Fig — GLT-1 (Texas Red, red) and GFAP (FITC, green) double staining of brain sections of animals subjected to sham surgery, preceded by preconditioning. Scale bars represent 25 μm, for each group n = 8. (TIF) [file pone.0186243.s001.tif]

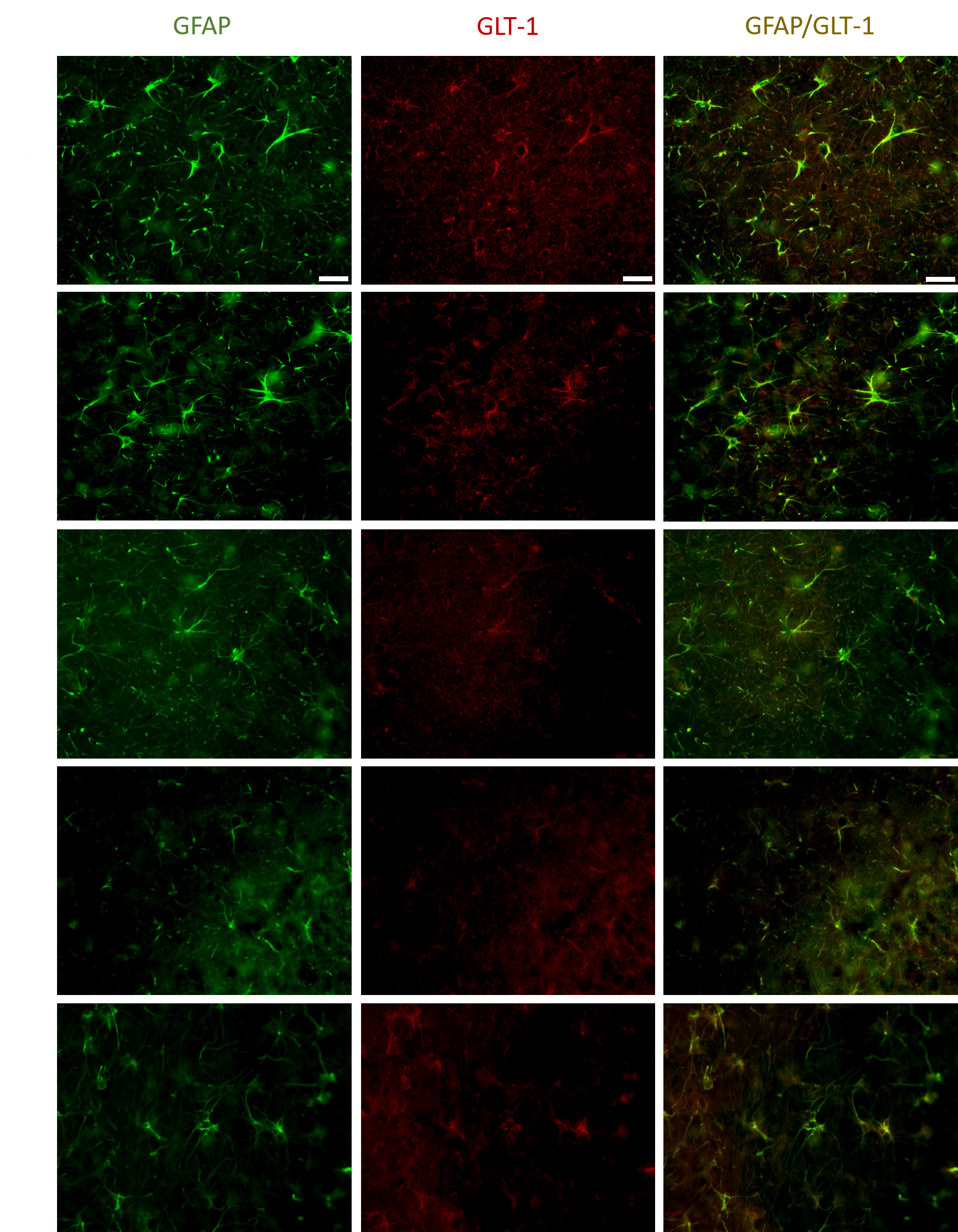

Supplement: S2 Fig — GLT-1 (Texas Red, red) and GFAP (FITC, green) double staining of brain sections of animals subjected to sham surgery, preceded by preconditioning. Scale bars represent 25 μm, for each group n = 8. (TIF) [file pone.0186243.s002.tif]

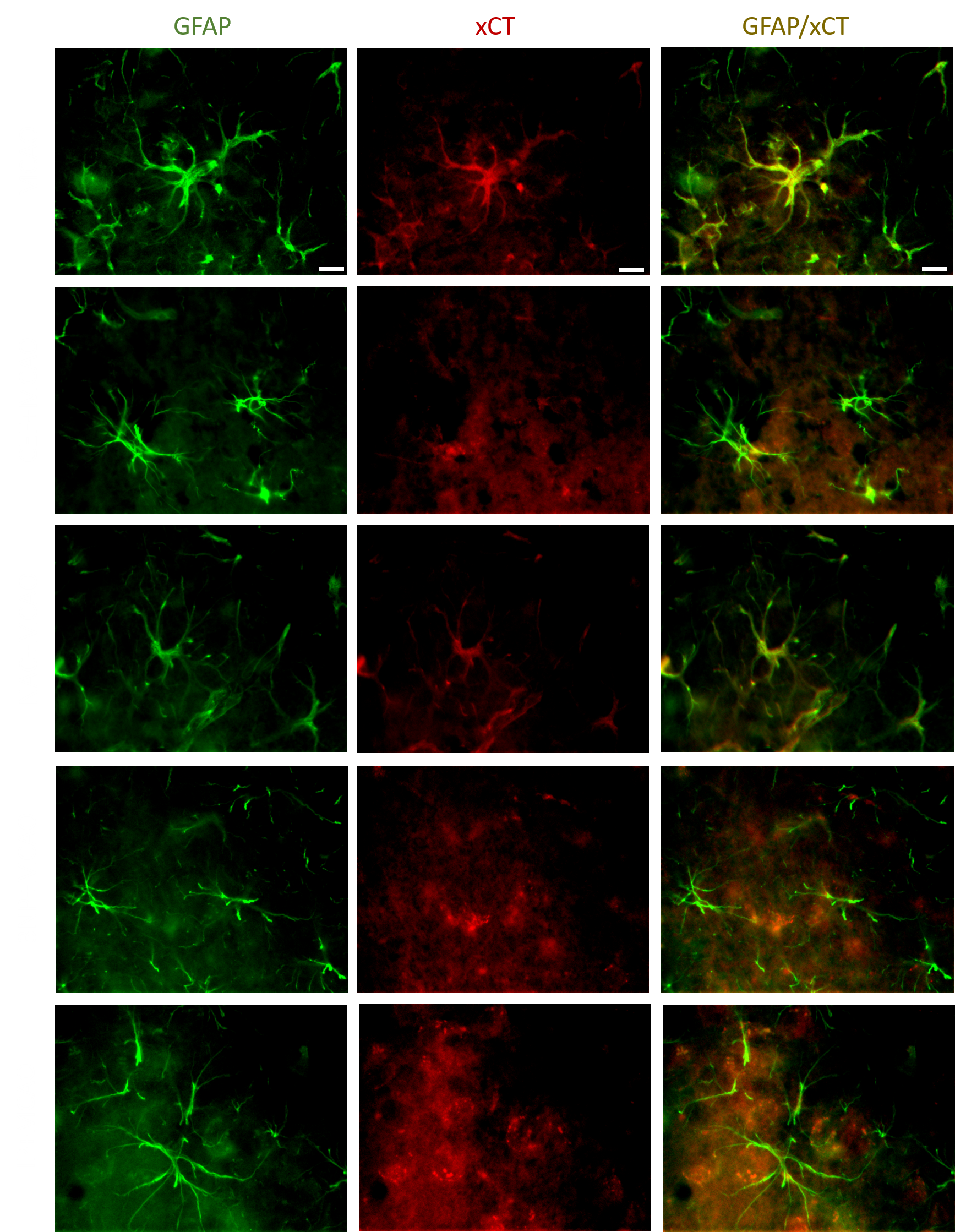

Supplement: S3 Fig — xCT (Texas Red, red) and GFAP (FITC, green) double staining of brain sections of animals subjected to 90-minute MCAO, preceded by preconditioning. Scale bars represent 10 μm, for each group n = 8. (TIF) [file pone.0186243.s003.tif]

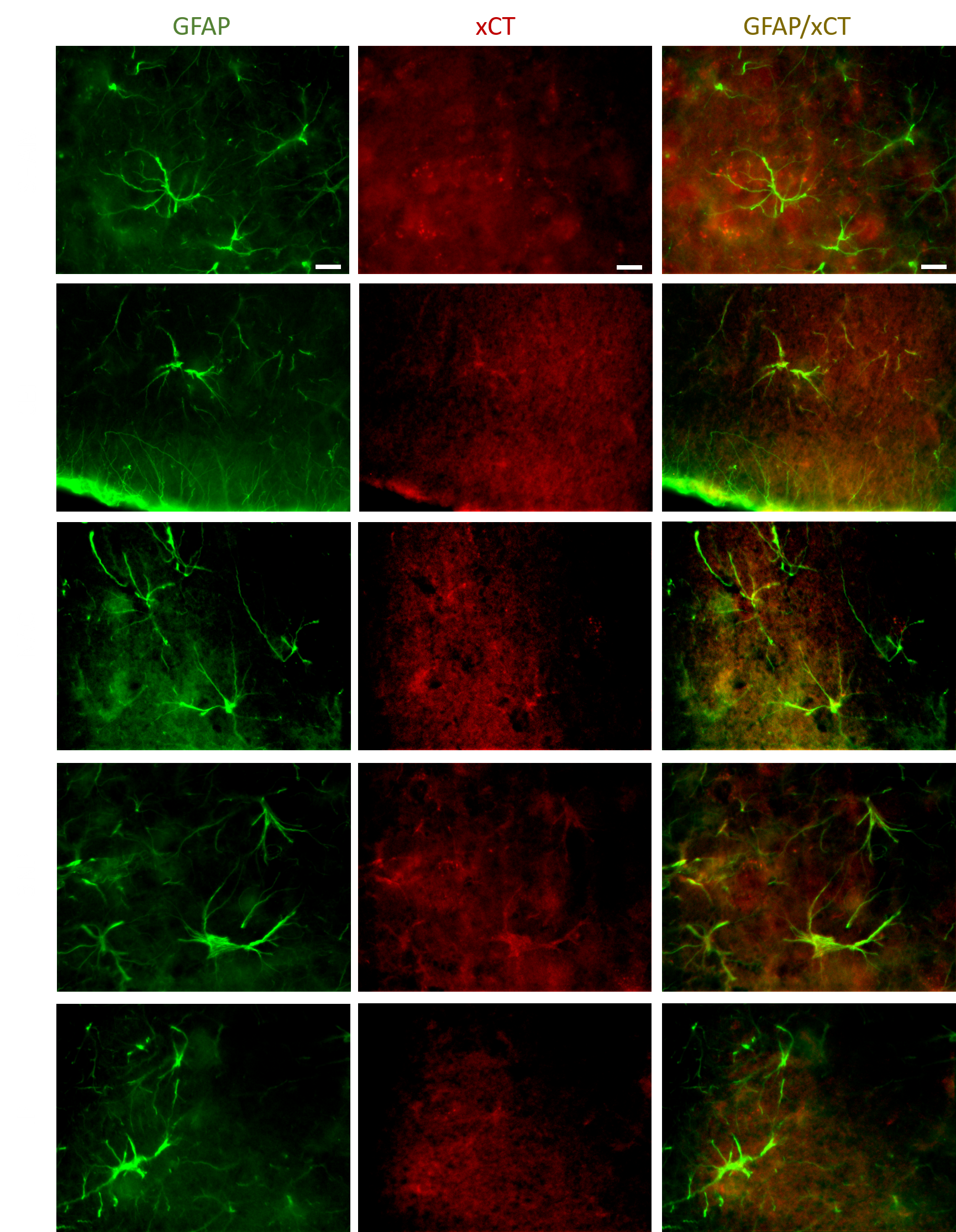

Supplement: S4 Fig — xCT (Texas Red, red) and GFAP (FITC, green) double staining of brain sections of animals subjected to sham surgery, preceded by preconditioning. Scale bars represent 10 μm, for each group n = 8. (TIF) [file pone.0186243.s004.tif]

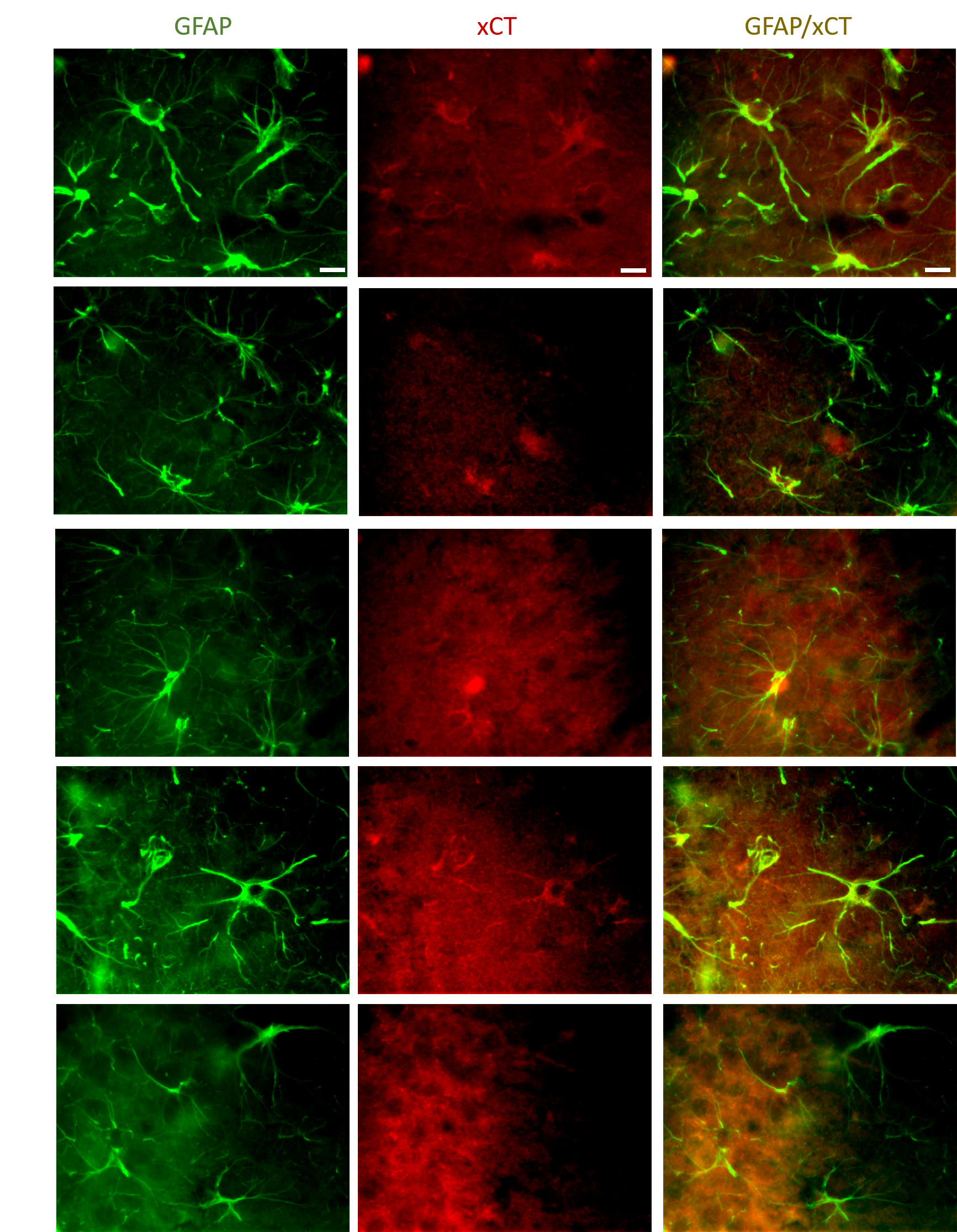

Supplement: S5 Fig — xCT (Texas Red, red) and GFAP (FITC, green) double staining of brain sections of animals subjected to 90-minute MCAO, preceded by preconditioning. Scale bars represent 10 μm, for each group n = 8. (TIF) [file pone.0186243.s005.tif]

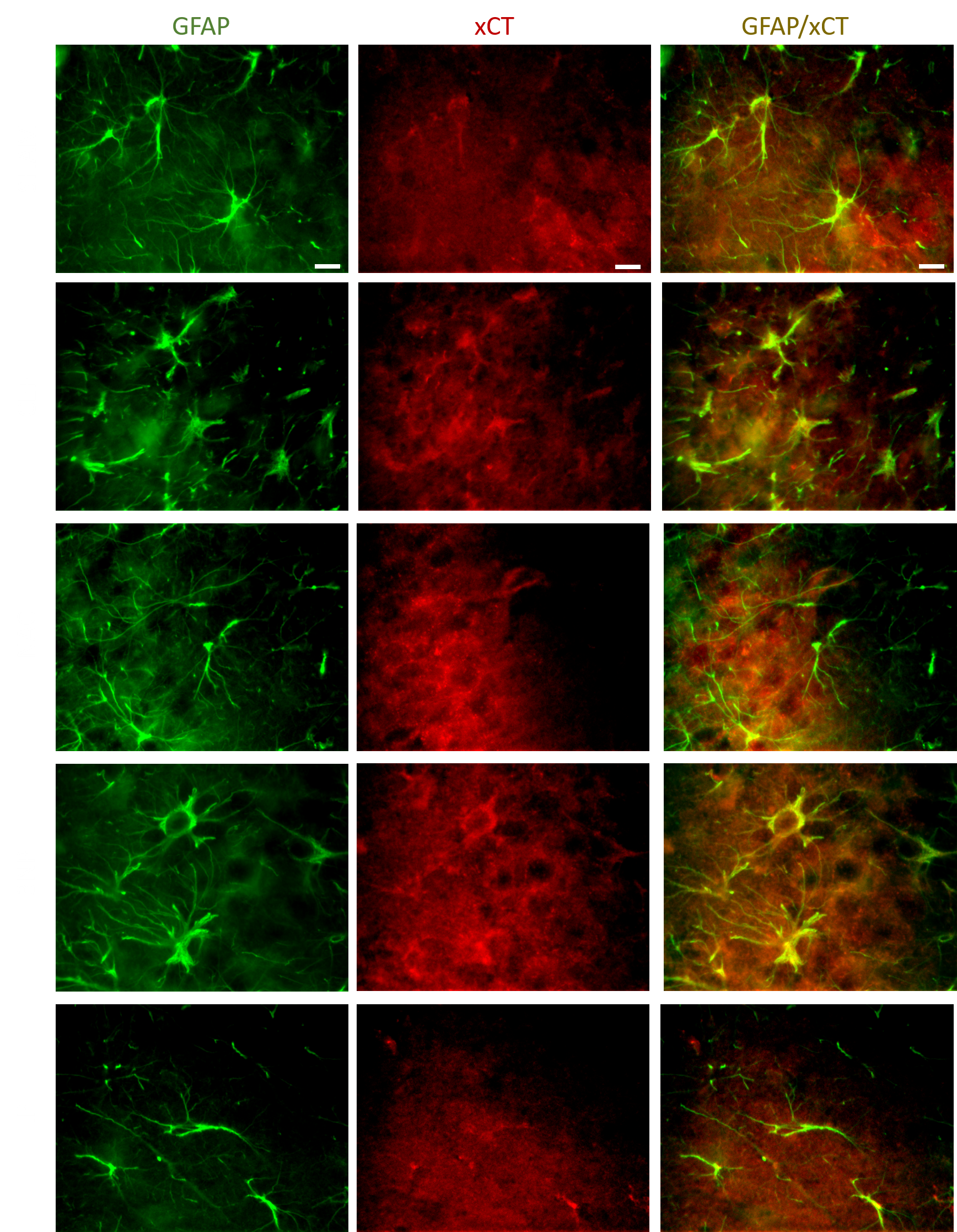

Supplement: S6 Fig — xCT- (Texas Red, red) and GFAP (FITC, green) double staining of brain sections of animals subjected to sham surgery, preceded by preconditioning. Scale bars represent 10 μm, for each group n = 8. (TIF) [file pone.0186243.s006.tif]

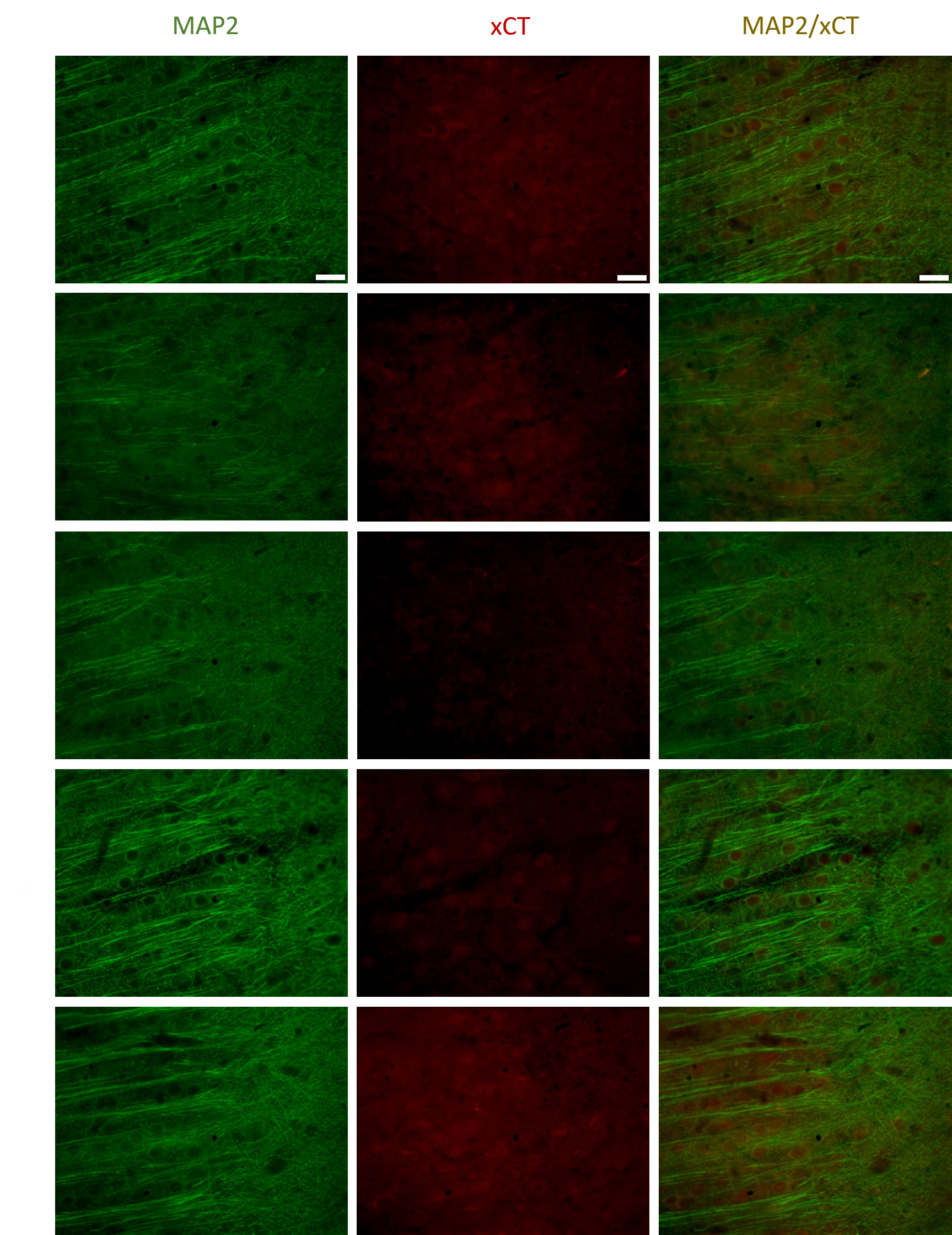

Supplement: S7 Fig — xCT (Texas Red, red) and MAP2 (Alexa Fluor 488, green) double staining of brain sections of animals subjected to sham surgery, preceded by preconditioning. Scale bars represent 25 μm, for each group n = 8. (TIF) [file pone.0186243.s007.tif]

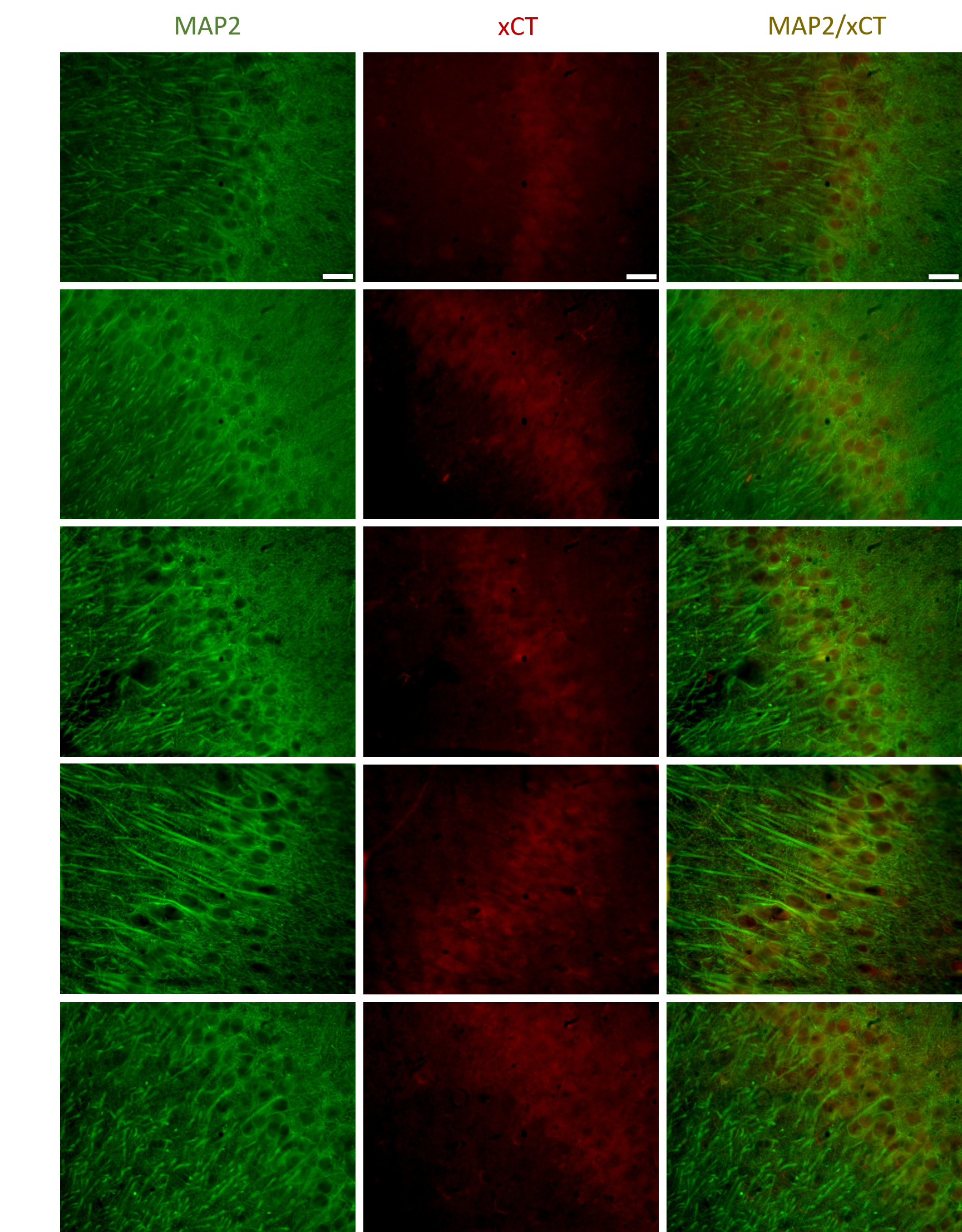

Supplement: S8 Fig — xCT (Texas Red, red) and MAP2 (Alexa Fluor 488, green) double staining of brain sections of animals subjected to sham surgery, preceded by preconditioning. Scale bars represent 25 μm, for each group n = 8. (TIF) [file pone.0186243.s008.tif]

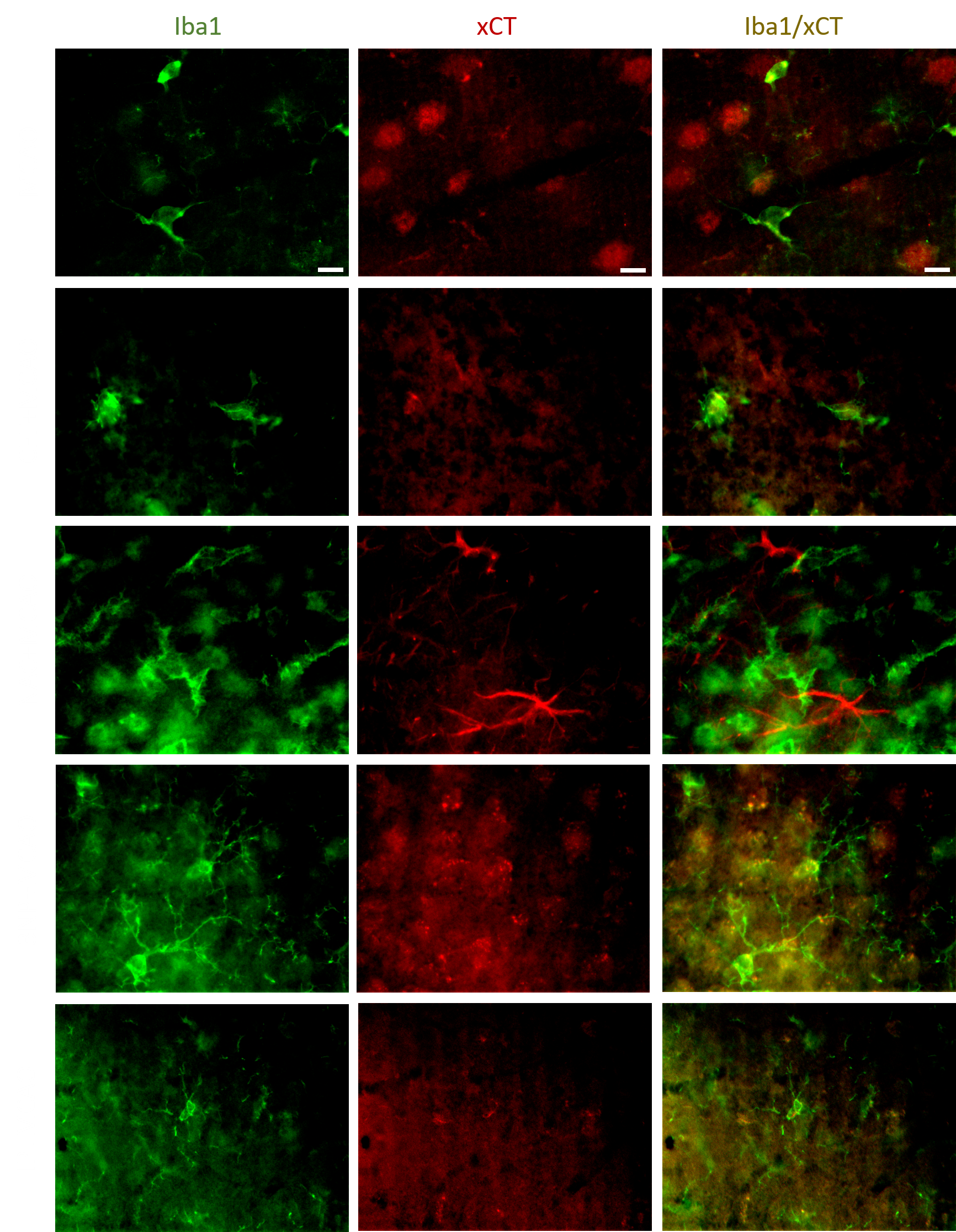

Supplement: S9 Fig — xCT (Texas Red, red) and Iba1 (FITC, green) double staining of brain sections of animals subjected to 90-minute MCAO, preceded by preconditioning. Scale bars represent 10 μm, for each group n = 8. (TIF) [file pone.0186243.s009.tif]

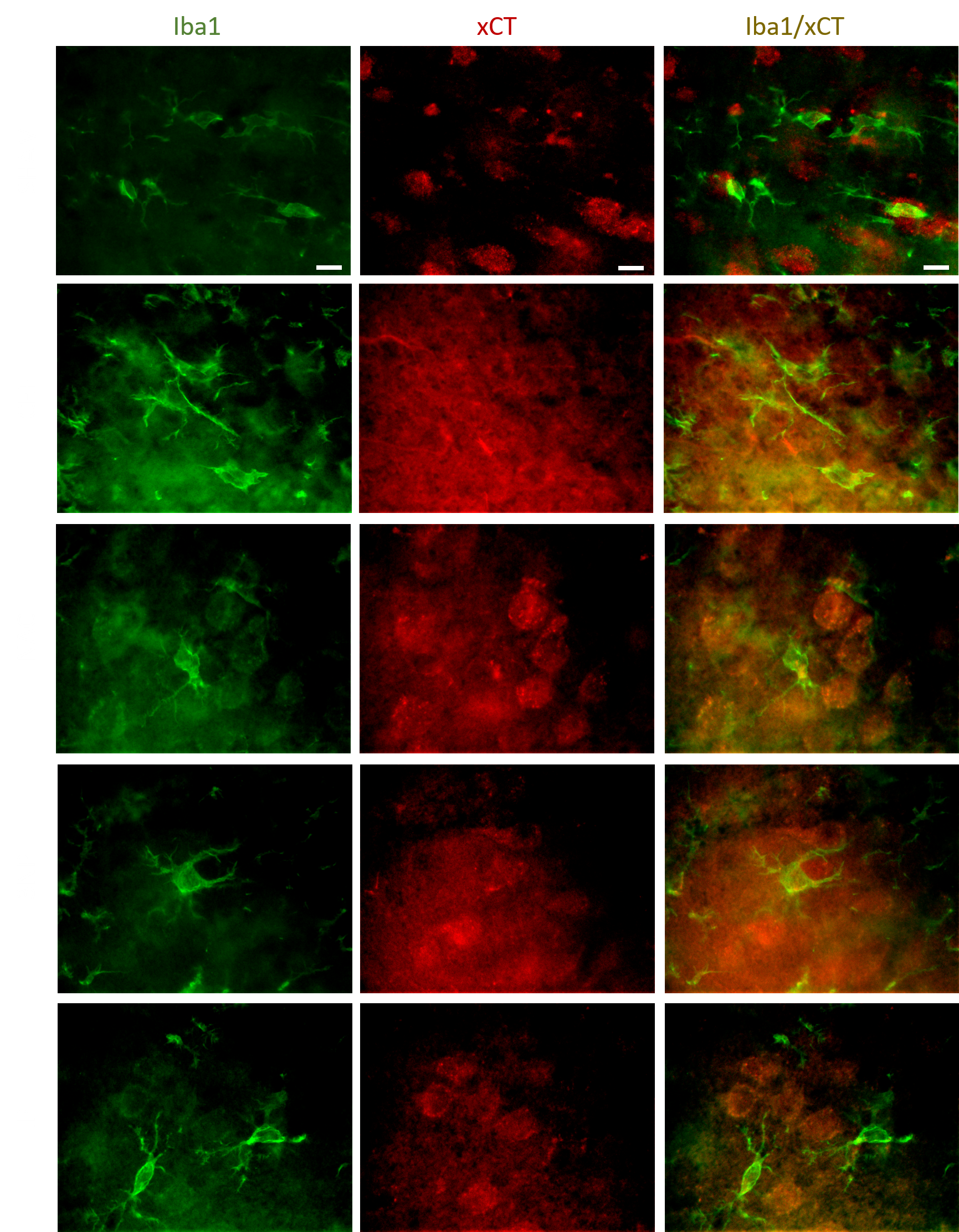

Supplement: S10 Fig — xCT (Texas Red, red) and Iba1 (FITC, green) double staining of brain sections of animals subjected to sham surgery, preceded by preconditioning. Scale bars represent 10 μm, for each group n = 8. (TIF) [file pone.0186243.s010.tif]

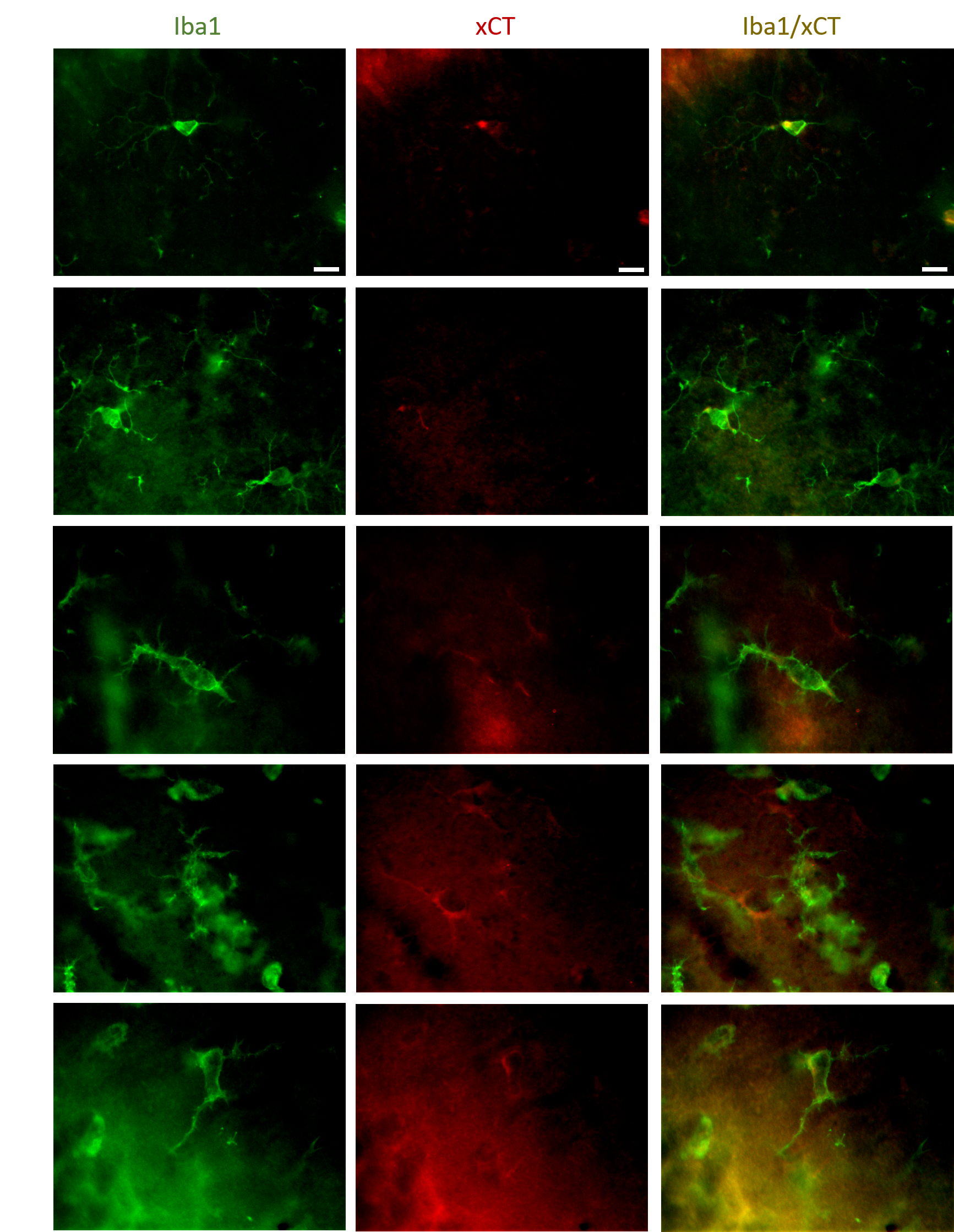

Supplement: S11 Fig — xCT (Texas Red, red) and Iba1 (FITC, green) double staining of brain sections of animals subjected to 90-minute MCAO, preceded by preconditioning. Scale bars represent 10 μm, for each group n = 8. (TIF) [file pone.0186243.s011.tif]

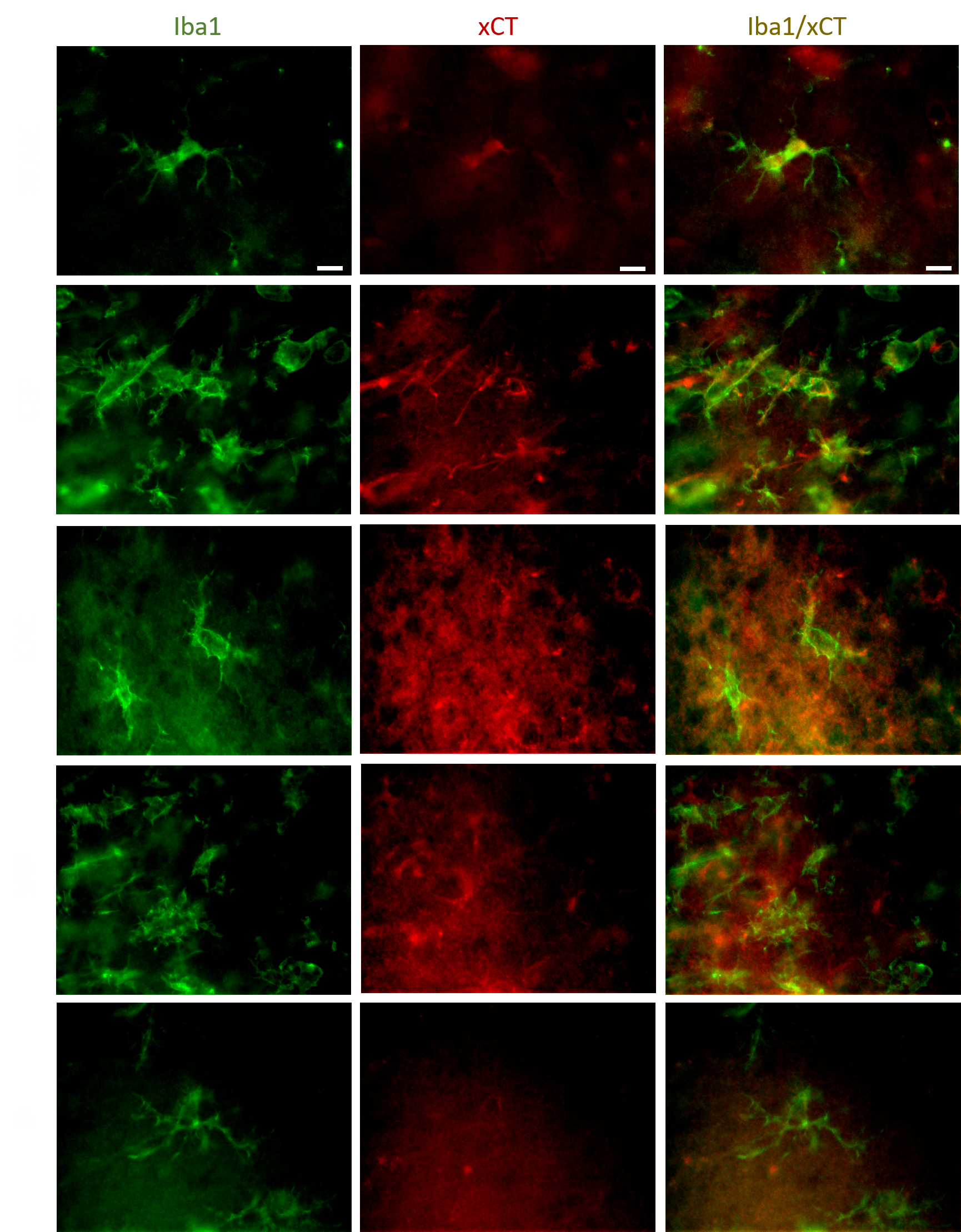

Supplement: S12 Fig — xCT (Texas Red, red) and Iba1 (FITC, green) double staining of brain sections of animals subjected to sham surgery, preceded by preconditioning. Scale bars represent 10 μm, for each group n = 8. (TIF) [file pone.0186243.s012.tif]

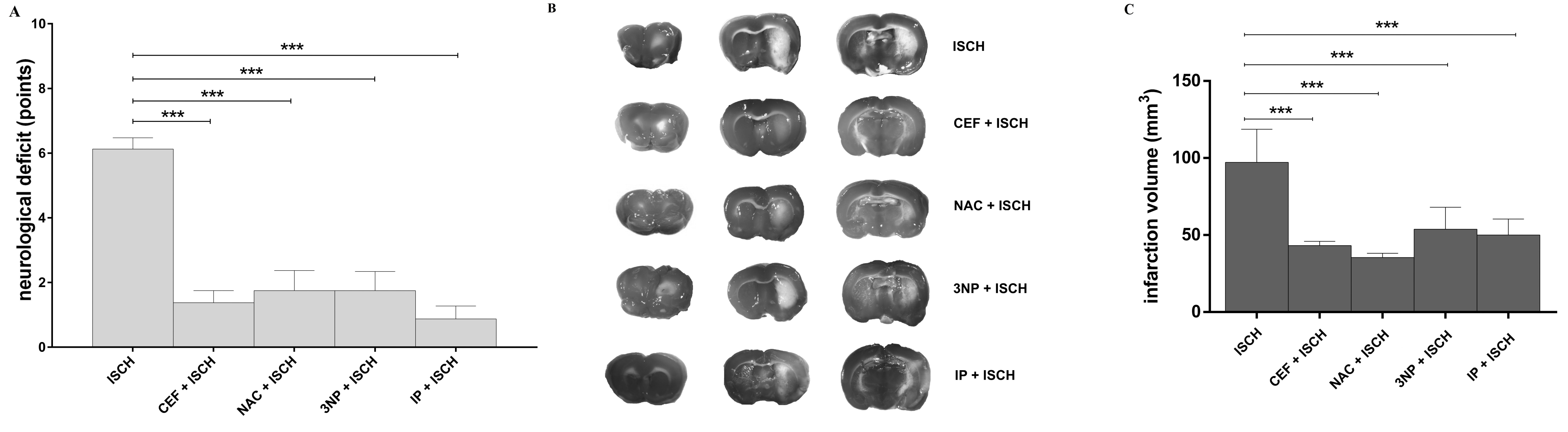

Supplement: S13 Fig — (A) decreased neurological deficit 24 h after ischemia (***p<0.001 vs. ISCH, Mann-Whitney, n = 24/group). (B) representative TTC-stained brain sections and corresponding histogram (C) representing calculated infarct volume in VEH+ISCH, CEF+ISCH, NAC+ISCH, 3NP+ISCH, and IP+ISCH groups (***p<0.001 vs. VEH+ISCH, t-test, n = 8/group) 24 h after reperfusion. The figure obtained from our previous manuscript entitled: ‘N-Acetylcysteine and Ceftriaxone as Preconditioning Strategies in Focal Brain Ischemia: Influence on Glutamate Transporters Expression’, Neurotoxicity Research, 2016; 29:539–550 under the terms of the Creative Commons Attribution 4.0 International License (http://creativecommons.org/licenses/by/4.0/). (TIF) [file pone.0186243.s013.tif]

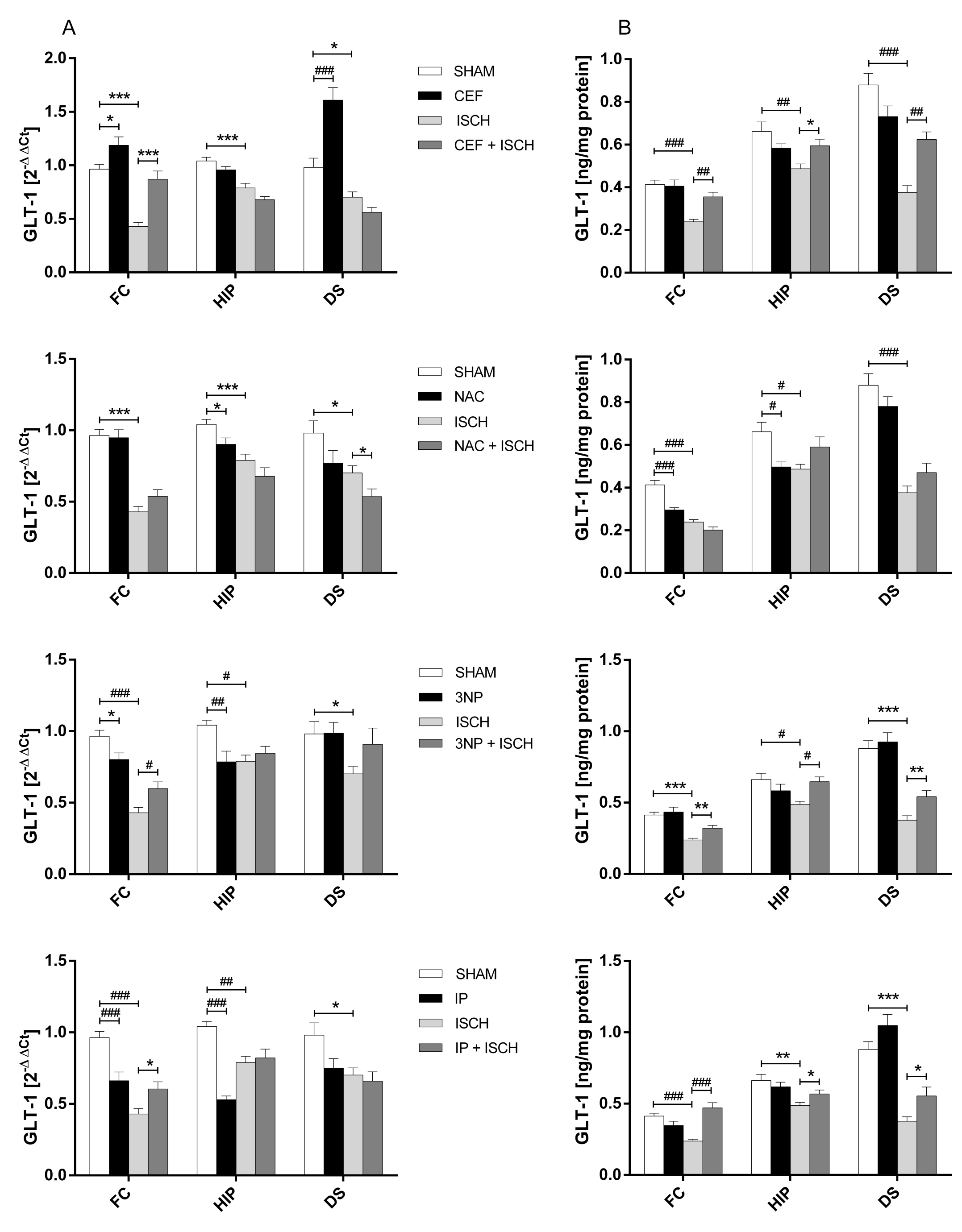

Supplement: S14 Fig — The figure obtained from our previous manuscript entitled: ‘N-Acetylcysteine and Ceftriaxone as Preconditioning Strategies in Focal Brain Ischemia: Influence on Glutamate Transporters Expression’, Neurotoxicity Research, 2016; 29:539–550 under the terms of the Creative Commons Attribution 4.0 International License (http://creativecommons.org/licenses/by/4.0/). (TIF) [file pone.0186243.s014.tif]

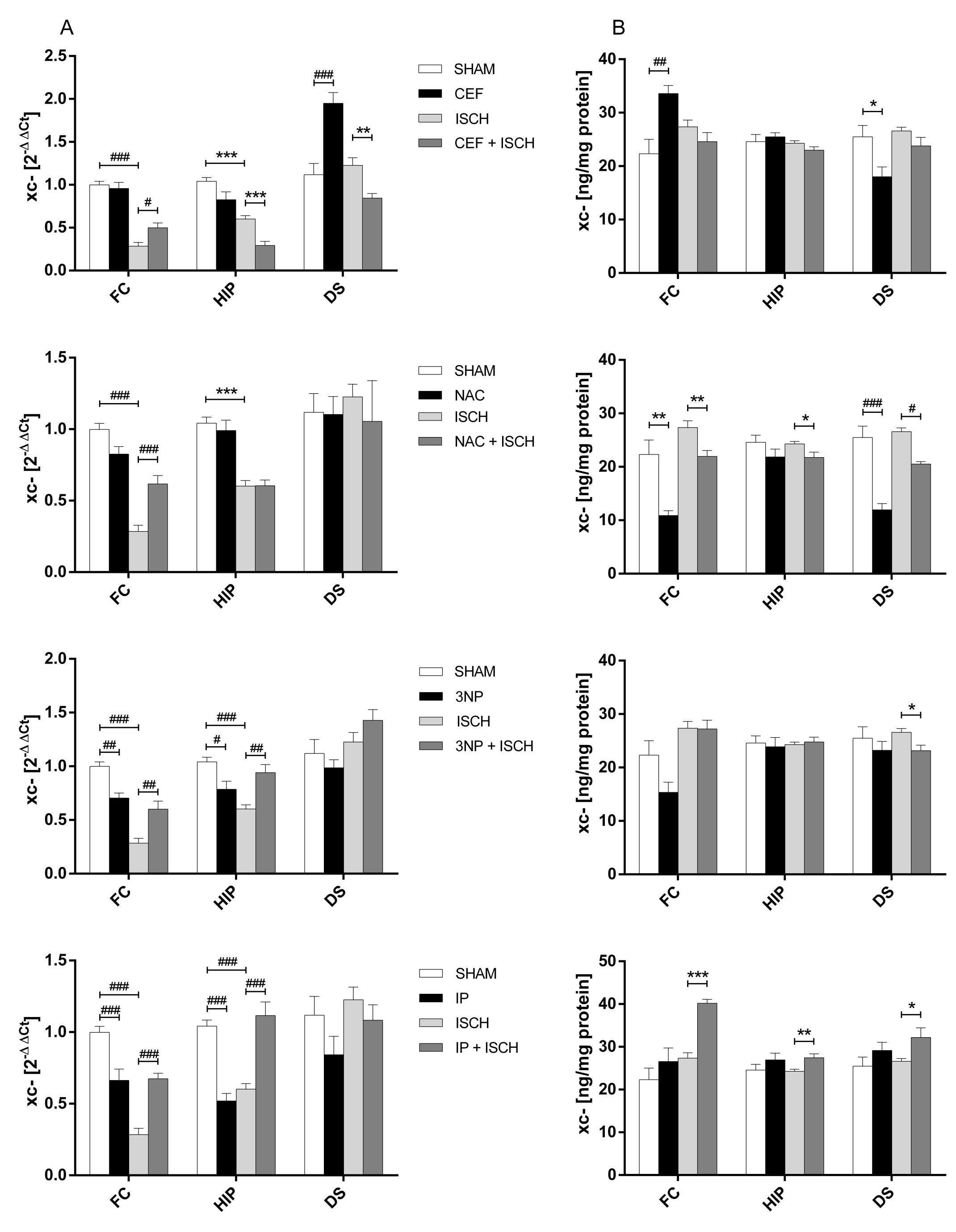

Supplement: S15 Fig — The figure obtained from our previous manuscript entitled: ‘N-Acetylcysteine and Ceftriaxone as Preconditioning Strategies in Focal Brain Ischemia: Influence on Glutamate Transporters Expression’, Neurotoxicity Research, 2016; 29:539–550 under the terms of the Creative Commons Attribution 4.0 International License (http://creativecommons.org/licenses/by/4.0/). (TIF) [file pone.0186243.s015.tif]

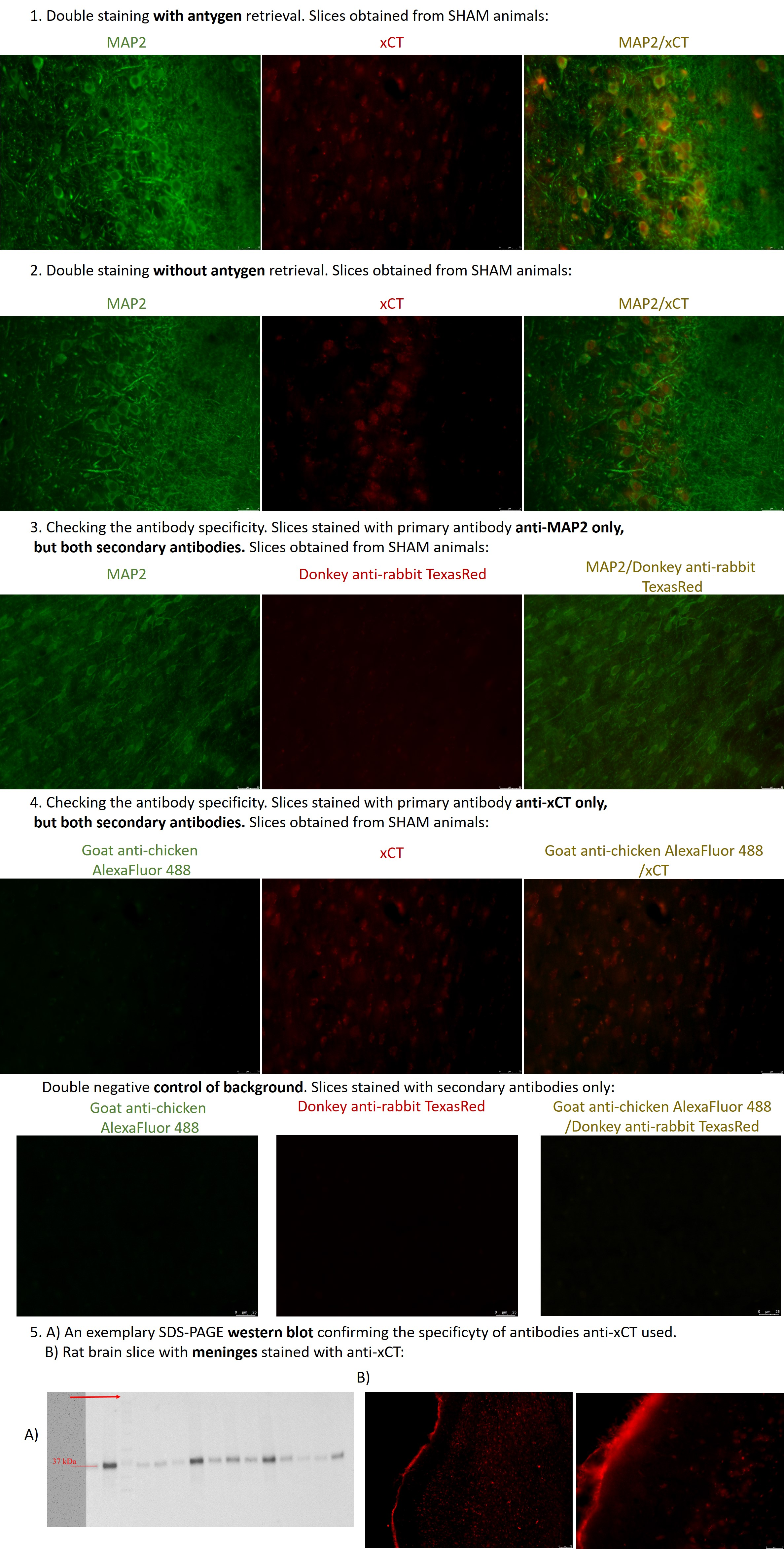

Supplement: S16 Fig — 1 & 2: double immunofluorescent staining with or without antigen retrieval. 3 & 4: negative staining control with the omission in the procedure adding of primary antibody against xCT, MAP2 or both antibodies. 5A: An exemplary immunoblot membrane obtained using the same antibody anti-xCT as in immunofluorescent staining. 5B: A positive control staining of brain meninges, where expression of system xc- is known to be high. (TIF) [file pone.0186243.s016.tif]
